# Spatiotemporal dissection of the *trans*-Golgi network in budding yeast

**DOI:** 10.1242/jcs.231159

**Published:** 2019-08-02

**Authors:** Takuro Tojima, Yasuyuki Suda, Midori Ishii, Kazuo Kurokawa, Akihiko Nakano

**Affiliations:** 1 Live Cell Super-Resolution Imaging Research Team, RIKEN Center for Advanced Photonics, Wako, Saitama 351-0198, Japan; 2 Laboratory of Molecular Cell Biology, Faculty of Medicine, University of Tsukuba, Tsukuba, Ibaraki 305-8575, Japan

**Keywords:** *Trans*-Golgi network, Membrane traffic, Cisternal maturation, Super-resolution live imaging, *Saccharomyces cerevisiae*

## Abstract

The *trans*-Golgi network (TGN) acts as a sorting hub for membrane traffic. It receives newly synthesized and recycled proteins, and sorts and delivers them to specific targets such as the plasma membrane, endosomes and lysosomes/vacuoles. Accumulating evidence suggests that the TGN is generated from the *trans*-most cisterna of the Golgi by maturation, but the detailed transition processes remain obscure. Here, we examine spatiotemporal assembly dynamics of various Golgi/TGN-resident proteins in budding yeast by high-speed and high-resolution spinning-disk confocal microscopy. The Golgi–TGN transition gradually proceeds via at least three successive stages: the ‘Golgi stage’ where glycosylation occurs; the ‘early TGN stage’, which receives retrograde traffic; and the ‘late TGN stage’, where transport carriers are produced. During the stage transition periods, earlier and later markers are often compartmentalized within a cisterna. Furthermore, for the late TGN stage, various types of coat/adaptor proteins exhibit distinct assembly patterns. Taken together, our findings characterize the identity of the TGN as a membrane compartment that is structurally and functionally distinguishable from the Golgi.

This article has an associated First Person interview with the first author of the paper.

## Abstract (precis)

**Highlighted Article:** The TGN displays two sub-stages of maturation: ‘early TGN’, when retrograde traffic is received, and ‘late TGN’, when transport carriers are produced. At the late TGN, various coat/adaptor proteins exhibit distinct assembly dynamics.

## INTRODUCTION

The Golgi and the *trans*-Golgi network (TGN) play a central role in membrane traffic in almost all eukaryotic cells (Glick and Nakano, 2009). The Golgi consists of a series of flattened membrane sacs called cisternae, which can be classified into several sub-compartments, *cis*, medial and *trans* cisternae, whereas the TGN is a tubular-reticular membrane network that is joined to the *trans*-face of the Golgi. Newly synthesized proteins that departed from the endoplasmic reticulum (ER) enter the Golgi at the *cis* cisterna and move progressively through medial and *trans* cisternae, and then reach the TGN (Suda et al., 2018). A major role of the Golgi is processing of the cargo proteins, whereas the TGN mediates sorting and packaging of the processed cargoes into transport carriers destined for individual intracellular and extracellular destinations (Paczkowski et al., 2015; De Matteis and Luini, 2008; Guo et al., 2014).

Earlier genetic and biochemical studies have identified a variety of coat and adaptor proteins responsible for the formation of transport carriers at the TGN. The coat protein clathrin and its adaptors, adaptor protein 1 (AP-1) complex and Golgi-localizing, γ-adaptin ear homology domain, Arf-binding (GGA) proteins, mediate budding of transport vesicles for the delivery to endosomes (Traub, 2005). Adaptor protein 3 (AP-3) complex mediates direct transport to lysosomes/vacuoles (Cowles et al., 1997; Vowels and Payne, 1998). The exomer complex is involved in direct transport of a subset of cargoes to the plasma membrane in yeast (Wang et al., 2006; Huranova et al., 2016). However, little is known so far about when and how these coat/adaptor proteins assemble at the TGN to coordinate selective cargo sorting and packaging.

Another important function of the TGN is the reception of retrograde membrane traffic from the endocytic pathway (Pavelka et al., 1998; Malsam and Sollner, 2011; Bonifacino and Rojas, 2006). This process includes tethering and subsequent fusion of endosome-derived vesicles with the TGN membrane (Graham, 2004). In addition, recent studies have shown that the TGN functions like early endosomes (Uemura and Nakano, 2013; Rosquete et al., 2018; Day et al., 2018). In particular, the plant TGN appears to be an independent organelle, rather than a sub-compartment of the Golgi (Uemura and Nakano, 2013; Dettmer et al., 2006). The biogenesis of the TGN is dependent on the dynamics of the Golgi. Accumulating evidence indicates that the cargo traffic through the Golgi/TGN is mediated by cisternal maturation, that is, a single cisterna gradually changes its nature from an earlier to a later one, while keeping the cargo proteins inside (Matsuura-Tokita et al., 2006; Losev et al., 2006; Glick and Luini, 2011; Nakano and Luini, 2010; Kurokawa et al., 2019; Casler et al., 2019). In this view, the TGN should be generated progressively from *trans*-Golgi cisternae by maturation, making it difficult to delineate a clear boundary between the Golgi and the TGN. Indeed, *trans*-Golgi cisterna and the TGN are often categorized together as ‘late Golgi’, and Sec7, an Arf1-guanine nucleotide exchange factor (GEF) in yeast, has often been used as a typical marker for ‘late Golgi’.

In order to characterize the identity of the TGN in the present study, we examined the spatiotemporal transition dynamics from the Golgi to the TGN through a super-resolution confocal live imaging microscopy (SCLIM) technique that we developed (Kurokawa et al., 2013; Matsuura-Tokita et al., 2006). We take advantage of the unstacked and dispersed nature of Golgi/TGN cisternae in the budding yeast *Saccharomyces cerevisiae* (Mowbrey and Dacks, 2009) to assess the transition dynamics. Through fluorescent protein tags, we visualized the following Golgi/TGN-resident proteins: Sec7, an Arf1 GEF which mediates carrier formation (Casanova, 2007; Richardson et al., 2012); Clc1, a clathrin light chain (Traub, 2005); Apl2, a component of the clathrin adaptor complex AP-1; Gga2, a GGA protein that acts as another clathrin adaptor (Black and Pelham, 2000; Zhdankina et al., 2001); Apl6, a component of the clathrin-independent adaptor AP-3 complex (Cowles et al., 1997; Vowels and Payne, 1998); Chs5, a component of the exomer complex (Wang et al., 2006; Huranova et al., 2016); Tlg2, a target soluble *N*-ethylmaleimide-sensitive fusion attachment protein receptor (t-SNARE) implicated in mediating fusion of endosome-derived vesicles with late Golgi (Abeliovich et al., 1998; Chen et al., 2010; Holthuis et al., 1998); Sys1, a late Golgi membrane protein that recruits the GRIP-domain golgin Imh1 via Arl1 and Arl3 GTPases (Behnia et al., 2004); Ypt6, the yeast Rab6 homolog that recruits the Golgi-associated retrograde protein (GARP) complex to regulate the fusion of endosome-derived vesicles with late Golgi (Siniossoglou and Pelham, 2001; Luo and Gallwitz, 2003); Sec21, a component of the coat protein complex I (COPI) that mediates intra-Golgi and Golgi-ER retrograde traffic (Ishii et al., 2016; Papanikou et al., 2015; Jackson, 2014); and Gnt1, a Golgi-resident glycosyltransferase (Yoko-o et al., 2003; Ishii et al., 2016). Our SCLIM observations at high spatiotemporal resolution demonstrate that the yeast TGN can be clearly distinguished from earlier Golgi cisternae based on its precise transition dynamics. Furthermore, the TGN can be divided into two sequential sub-stages: ‘early TGN’, which receives retrograde traffic and ‘late TGN’, which produces transport carriers. We also find that, at the late TGN stage, individual coat/adaptor proteins exhibit distinct assembly dynamics.

## RESULTS

### 3D distribution of various Golgi/TGN-resident proteins

First, we performed 3D (*xyz*) colocalization analysis of Golgi/TGN-resident proteins by dual-color SCLIM imaging (Fig. S1A–J). We tagged Clc1, Gga2, Apl2, Apl6, Chs5, Tlg2, Sys1 and Sec21 with GFP, and Sec7 with tagRFP or mCherry. Here, Sec7 was used as a reference marker for the late Golgi (the *trans*-Golgi, TGN or both). We found that all the examined proteins were mainly distributed as many punctate compartments (∼1 µm in diameter) within a cell. The degree of colocalization versus Sec7 was high for Clc1, Gga2, Apl2 and Chs5 [Pearson's correlation coefficient (*r*)>0.62] (Fig. S1A–C,E,J), moderate for Tlg2 and Sys1 (0.55<*r*<0.57) (Fig. S1F,G,J), and low for Apl6 and Sec21 (*r*<0.54) (Fig. S1D,H,J). As a positive control experiment, we found almost perfect colocalization between Sec7–GFP and Sec7–tagRFP (*r*=0.92) (Fig. S1I,J). A simple hypothesis explaining such partial colocalization is that the examined GFP-tagged proteins reside not only in Sec7-containing compartments but also other organelles, such as endosomes. Alternatively, based on the concept of cisternal maturation, the partial colocalization could reflect the temporal status of Golgi/TGN maturation. Namely, if the timing of recruitment of the two proteins to a single cisterna is not perfectly synchronized, the cisterna would harbor only either one of them at some time-points of maturation. To examine this possibility, we next performed 4D (*xyz* plus time) dual-color SCLIM imaging.

### Clathrin assembles on late phase Sec7-containing cisternae

**Fig. 1.**
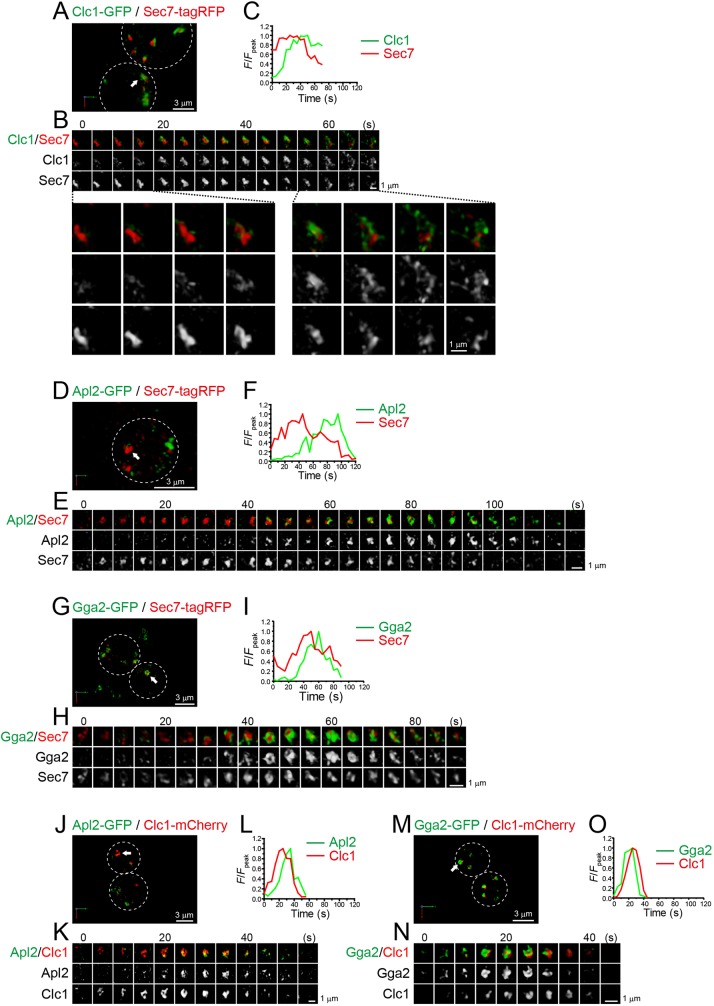
**4D dynamics of clathrin** **and its adaptors, AP-1 and GGA proteins, at the Sec7**-**containing** **compartment.** Dual-color time-lapse SCLIM imaging of yeast cells expressing Clc1–GFP (a clathrin subunit) and Sec7–tagRFP (A–C), Apl2–GFP (an AP-1 subunit) and Sec7–tagRFP (D–F), Gga2–GFP (a GGA protein) and Sec7–tagRFP (G–I), Apl2–GFP and Clc1–mCherry (J–L), and Gga2–GFP and Clc1–mCherry (M–O). (A,D,G,J,M) Low-magnification images of the cells. The white broken lines indicate the edge of the cells. (B,E,H,K,N) Time-lapse images of the single cisternae (white arrows in A, D, G, J and M, respectively) in the cells. Lower panels in B show magnified images at the selected time points. (C,F,I,L,O) Time course changes in relative fluorescence intensities (*F*/*F*_peak_) of green and red channels in B, E, H, K and N, respectively. Scale bars: 3 µm (A,D,G,J,M) and 1 µm (B,E,H,K,N).

Clathrin, a major coat protein complex consisting of light and heavy chains (Clc1 and Chc1, respectively), has been thought to mediate budding of transport vesicles between the TGN and endosomes (Robinson, 2015). In addition, a recent paper presented another interesting hypothesis that clathrin and its adaptor AP-1 mediate retrograde recycling of TGN proteins (Day et al., 2018; Papanikou et al., 2015; Casler et al., 2019). We compared the dynamics of Clc1–GFP versus Sec7–tagRFP in live cells (Fig. 1A–C; Fig. S2A). Consistent with previous reports (Matsuura-Tokita et al., 2006; Losev et al., 2006), 4D SCLIM imaging showed many mobile Sec7–tagRFP-containing compartments appearing and disappearing with a lifetime of a few minutes. Dual-color 4D imaging showed that the majority of Clc1–GFP signals appeared at pre-existing Sec7-containing compartments (Fig. 1B). The fluorescence intensity of the Sec7–tagRFP signals peaked earlier than that of Clc1–GFP (Fig. 1C; Table S1). Magnified images (Fig. 1B, lower panels) show that, although experimental variation was high, Clc1–GFP signals initially appeared as several small dot structures (<0.2 µm in diameter) on a pre-existing Sec7–tagRFP-containing compartment, and increased gradually their volume to cover up almost the entire region of the compartment. These Clc1–GFP signals most likely represent clathrin-coated buds formed on the surface of the TGN. Subsequently, the accumulated Clc1–GFP signals fragmented into many tubules and/or small dot-like structures, and then scattered into the cytoplasm (Fig. 1B; Movie 1). The scattered dots could represent clathrin-coated vesicles budding off from the TGN. In parallel with this Clc1–GFP disassembly, the Sec7–tagRFP-positive compartment also decreased their volume and eventually became invisible. A previous study also showed that Sec7-labeled TGN compartments disintegrate into several smaller structures (McDonold and Fromme, 2014). These results suggest strongly that, at the final stage of cisternal maturation, the TGN cisternae disappear owing to fragmentation into many small carrier vesicles.

### Sequential assembly of GGA proteins and AP-1 at clathrin-containing cisternae

Two classes of clathrin adaptors, AP-1 complex and GGA proteins, have been shown to function at the yeast TGN (Black and Pelham, 2000; Zhdankina et al., 2001; Nakayama and Wakatsuki, 2003). AP-1 complex consists of four subunits, Apl2, Apl4, Apm4 and Aps1, whereas two GGA proteins, Gga1 and Gga2, act as monomeric adaptors. We compared the dynamics of Apl2–GFP versus Sec7–tagRFP (Fig. 1D–F; Fig. S2B; Table S1), and Gga2–GFP versus Sec7–tagRFP (Fig. 1G–I; Fig. S2C; Table S1), by dual-color 4D SCLIM. Like Clc1, both Apl2–GFP and Gga2–GFP signals transiently appeared at Sec7–tagRFP-containing compartments during the decay phase of Sec7.

We then analyzed temporal relationships of Apl2 and Gga2 versus Clc1 (Fig. 1J–O; Fig. S3; Table S1). Gga2–GFP and Apl2–GFP signals preferentially appeared at Clc1–mCherry-containing compartments during the rise and decay phases of Clc1, respectively. These results suggest strongly that GGA and AP-1 sequentially accumulate at the TGN to recruit the clathrin coat, consistent with a previous report (Daboussi et al., 2012).

### AP-3 appears as small dot-like structures at Sec7-containing cisternae

**Fig. 2.**
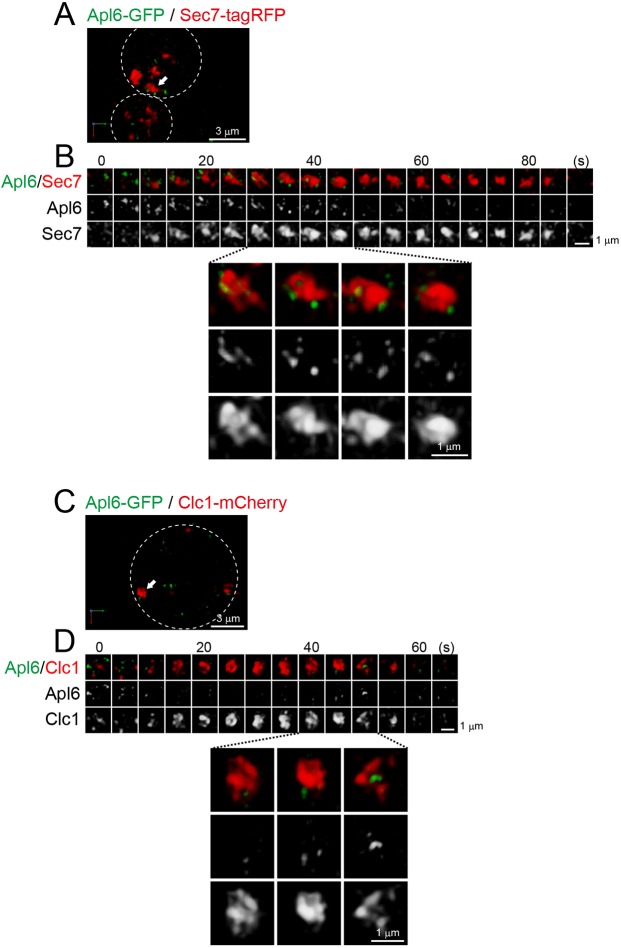
**4D dynamics of AP-3 at the Sec7**-**containing** **compartment.** Dual-color time-lapse SCLIM imaging of yeast cells expressing Apl6–GFP (an AP-3 subunit) and Sec7–tagRFP (A,B), and Apl6–GFP and Clc1–mCherry (C,D). (A,C) Low-magnification images of the cells. The white broken lines indicate the edge of the cells. (B,D) Upper panels show time-lapse images of the single cisternae (white arrows in A and C, respectively) in the cells. Lower panels show magnified images at the selected time-points. Scale bars: 3 µm (A,C) and 1 µm (B,D).

AP-3, another adaptor protein complex, is known to be involved in clathrin-independent traffic from the late Golgi to the vacuoles in yeast (Cowles et al., 1997; Vowels and Payne, 1998). It consists of four subunits, Apl6, Apl5, Apm3 and Aps3. Although 3D analysis revealed that Apl6–GFP and Sec7–tagRFP showed lower degrees of colocalization (Fig. S1D,J) (Day et al., 2018; Angers and Merz, 2009), we focused on the dynamics of Apl6–GFP at Sec7–tagRFP-containing compartments by dual-color 4D SCLIM (Fig. 2A,B; Fig. S4A). We found that, in the close proximity of a Sec7–tagRFP-containing compartment, Apl6–GFP signals appeared as multiple small dot structures (<0.2 µm in diameter) (Fig. 2B, lower panels; Movie 2). These small dots appeared at different times and remained stationary for several to a few tens of seconds without changing the size until they disappeared. Although a recent study also performed dual time-lapse imaging of AP-3 and Sec7 (Day et al., 2018), our advanced microscopy has succeeded, for the first time, in visualizing such a fine spatiotemporal dynamics of AP-3. The Apl6 dynamics could represent the formation and budding-off of AP-3-positive vesicles from the TGN. Notably, we occasionally observed that Apl6–GFP-positive dots already existed before the appearance of Sec7–tagRFP-containing compartment (see Fig. 2B, time-points 0 and 5 s), suggesting that AP-3 can assemble not only during stage at which Sec7 is resident but also at earlier Golgi stages.

We also visualized Apl6–GFP together with Clc1–mCherry, and found that several Apl6-positive dots were also located in the close proximity of a Clc1-containing compartment (Fig. 2C,D; Fig. S4B). This suggests that a single TGN cisterna can recruit multiple adaptors (AP-1, GGA and AP-3) with different spatiotemporal dynamics. In particular, the assembly pattern of AP-3 was distinct from those of AP-1 and GGA clathrin adaptors, consistent with the notion that AP-3 functions independently of clathrin in yeast (Vowels and Payne, 1998).

### Exomer and Sec7 show synchronized assembly dynamics

**Fig. 3.**
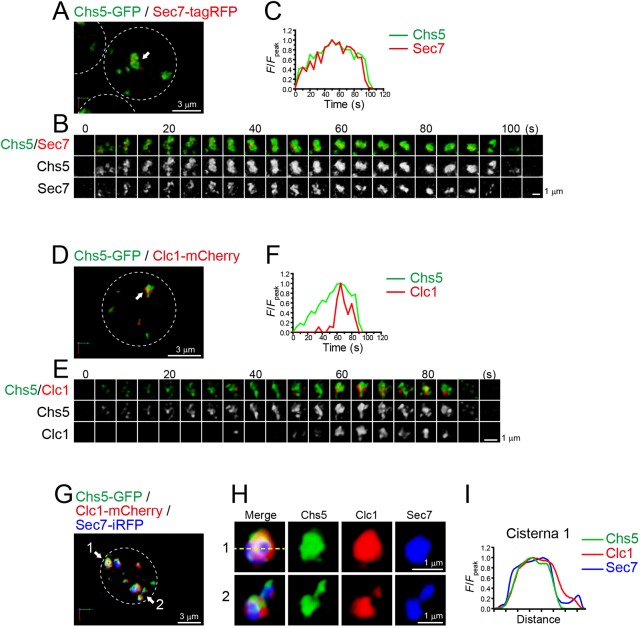
**4D and 3D dynamics of exomer at the Sec7**-**containing** **compartment.** (A–F) Dual-color time-lapse SCLIM imaging of yeast cells expressing Chs5–GFP (an exomer subunit) and Sec7–tagRFP (A–C), and Chs5–GFP and Clc1–mCherry (D–F). (A,D) Low-magnification images of the cells. (B,E) Time-lapse images of the single cisternae (white arrows in A and C, respectively) in the cells. (C,F) Time course changes in relative fluorescence intensities (*F*/*F*_peak_) of green and red channels in B and E, respectively. (G–I) Triple-color SCLIM imaging of yeast cells expressing Sec7–iRFP, Clc1–mCherry and Chs5–GFP. (G) Low-magnification images of the cells. (H) Magnified images of the two cisternae (1 and 2, white arrows in G) in the cell. (I) Line scan analysis of the cisterna 1 fluorescence. The *F*/*F*_peak_ values for the green, red and iRFP channels along the yellow broken line in H are profiled. The white broken lines in A, D and G indicate the edge of the cells. Scale bars: 3 µm (A,D,G) and 1 µm (B,E,H).

Exomer, a protein complex consisting of Chs5, Csh6, Bch1, Bch2 and Bud7, is involved in direct transport of a subset of cargo proteins from the TGN to the plasma membrane in yeast (Wang et al., 2006; Huranova et al., 2016). We examined the dynamics of Chs5–GFP versus Sec7–tagRFP by dual-color 4D SCLIM (Fig. 3A–C; Fig. S4C; Table S1). Consistent with the highest degree of colocalization with Sec7 in 3D analysis (Fig. S1), a time-course of the changes in Chs5–GFP fluorescence showed almost complete synchronization to those of Sec7–tagRFP. This suggests that the exomer-mediated cargo export occurred throughout the whole lifetime of the Sec7-containing compartment.

We also compared dynamics of Chs5–GFP versus Clc1–mCherry, and found that Clc1 mainly appeared at the decay phase of Chs5 (Fig. 3D–F; Fig. S4D; Table S1). To further confirm the co-existence of clathrin and exomer at a single TGN cisterna, we performed triple-color 3D SCLIM imaging (Fig. 3G–I; Movie 3). Both Clc1–mCherry and Chs5–GFP signals resided at a single Sec7–iRFP-containing compartment, and line scan analysis showed that their spatial distributions were very much alike (Fig. 3I).

Taken collectively, we demonstrated in Figs 1–3 that multiple types of coat and adaptor proteins (Clc1, AP-1, GGA, AP-3 and exomer) are recruited to the TGN in a spatially or temporally distinct manner.

### The t-SNARE Tlg2 accumulates immediately prior to Sec7

**Fig. 4.**
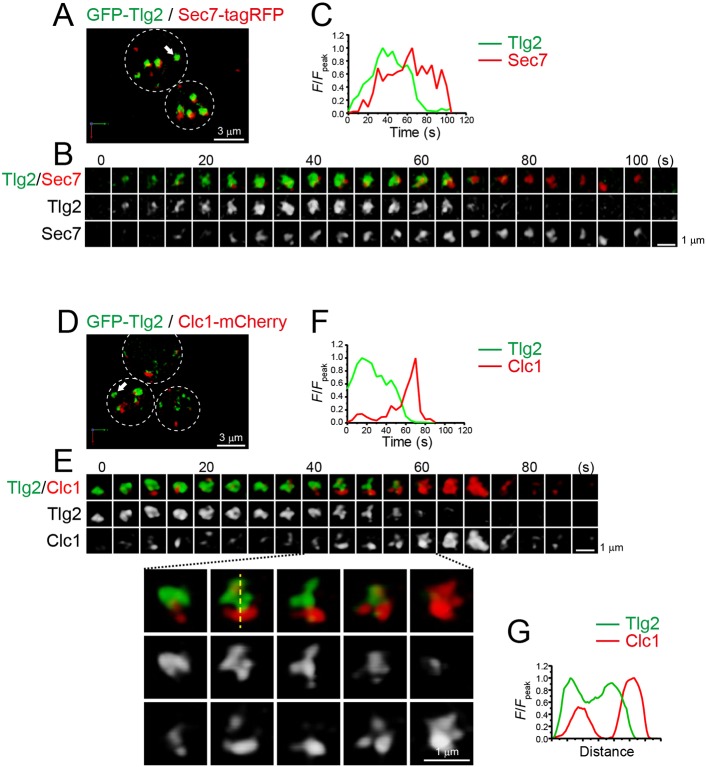
**4D dynamics of the t-SNARE Tlg2 at the Sec7**-**containing** **compartment.** Dual-color time-lapse SCLIM imaging of yeast cells expressing GFP–Tlg2 and Sec7–tagRFP (A–C), and GFP–Tlg2 and Clc1–mCherry (D–G). (A,D) Low-magnification images of the cells. The white broken lines indicate the edge of the cells. (B,E) Time-lapse images of the single cisternae (white arrows in A and D, respectively) in the cells. Lower panels in E show magnified images at the selected time-points. (C,F) Time course changes in relative fluorescence intensities (*F*/*F*_peak_) for the green and red channels in B and E, respectively. (G) Line scan analysis of GFP–Tlg2 and Clc1–mCherry signals in the single cisterna. The *F*/*F*_peak_ values (green and red channels) along the yellow broken line in E are profiled. Scale bars: 3 µm (A,D) and 1 µm (B,E).

In addition to the role in producing transport carriers (Figs 1–3), the TGN receives retrograde membrane traffic from endosomal pathways (Papanikou and Glick, 2014). This process includes tethering and subsequent fusion of endosome-derived vesicles with the TGN membrane (Graham, 2004). The vesicle fusion is mediated by a set of SNARE proteins including Tlg2, a late Golgi-resident t-SNARE (Abeliovich et al., 1998; Chen et al., 2010). We here examined the dynamics of Tlg2–GFP versus Sec7–tagRFP by dual-color 4D SCLIM imaging (Fig. 4A–C; Fig. S5A; Table S1). GFP–Tlg2 signals appeared first, and then Sec7–tagRFP signals appeared on the pre-existing GFP–Tlg2-containing compartment. The peak time-point of GFP–Tlg2 was earlier than that of Sec7–tagRFP. This suggests that Tlg2-mediated retrograde cargo reception precedes Sec7-mediated transport carrier formation.

We also analyzed GFP–Tlg2 dynamics versus Clc1–mCherry (Fig. 4D–G; Fig. S5B; Table S1). As expected, the GFP–Tlg2 signal appeared first, and then the Clc1–mCherry began to accumulate during the decay phase of GFP–Tlg2 signals. Magnified images (Fig. 4E, lower panels) showed that the transition from GFP–Tlg2 to Clc1–mCherry progressed via gradual decrease of the area of Tlg2-positive zone and complementary increase of the area of Clc1-positive zone. Notably, the two zones appeared to be segregated spatially (Fig. 4G). We have recently performed correlative light and electron microscopy (CLEM) and confirmed that, during Golgi cisternal maturation, earlier and later marker proteins are located within a continuous membrane structure in a segregated manner (Kurokawa et al., 2019). These findings strongly support the idea that a maturing Golgi/TGN cisterna includes discrete functional zones, for example, the retrograde cargo reception zone and the carrier formation zone.

### Sys1 accumulates prior to Tlg2

**Fig. 5.**
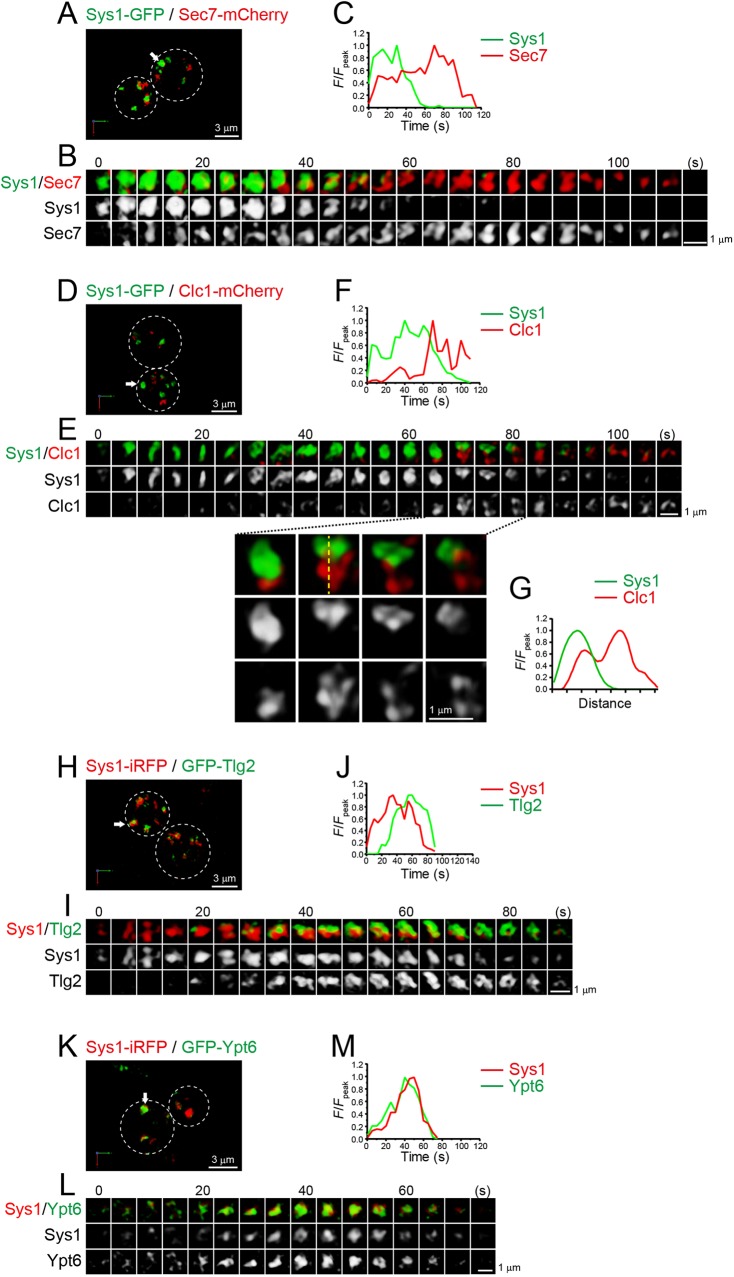
**4D dynamics of Sys1 versus Sec7, the t-SNARE Tlg2 and the Rab GTPase Ypt6.** Dual-color time-lapse SCLIM imaging of yeast cells expressing Sys1–GFP and Sec7–mCherry (A–C), Sys1–GFP and Clc1–mCherry (D–G), Sys1–iRFP and GFP–Tlg2 (H–J), and Sys1–iRFP and GFP–Ypt6 (K–M). (A,D,H,K) Low-magnification images of the cells. The white broken lines indicate the edge of the cells. (B,E,I,L) Time-lapse images of the single cisternae (white arrows in A, D, H and K, respectively) in the cells. (C,F,J,M) Time course changes in relative fluorescence intensities (*F*/*F*_peak_) for the green and red channels in B, E, I and L, respectively. Lower panels in E show magnified images at the selected time-points. (G) Line scan analysis of Sys1–GFP and Clc1–mCherry signals in the single cisterna. The *F*/*F*_peak_ values (green and red channels) along the yellow broken line in E are profiled. Scale bars: 3 µm (A,D,H,K) and 1 µm (B,E,I,L).

Sys1, an integral membrane protein that localizes to the late Golgi, is implicated in the recruitment of the GRIP-domain golgin Imh1 (Behnia et al., 2004). It binds to Arl3 GTPase on the surface of the late Golgi, and Arl3 then recruits Arl1, which in turn recruits Imh1 (Graham, 2004). Mammalian homologs of Imh1, such as golgin97 (also known as GOLGA1), are known to act as a putative tether that captures endosome-derived vesicles to promote their fusion with the TGN membrane (Munro, 2011), implicating a possible function of Sys1–Arl3–Arl1–Imh1 signaling cascade in the endosome–TGN traffic in yeast (Yu and Lee, 2017; Setty et al., 2004; Behnia et al., 2004). A previous paper showed that Sys1 and Sec7 exhibited almost the same temporal dynamics (Losev et al., 2006). However, we recently found that Sys1 came earlier than Sec7 (Ishii et al., 2016; Kurokawa et al., 2019) (Table S1). In the present study, we further examined the exact time-course of Sys1 recruitment relative to other Golgi/TGN markers, Sec7, Clc1 and Tlg2 (Fig. 5; Fig. S6; Table S1). Sys1–GFP preceded Sec7–mCherry (Fig. 5A–C; Fig. S6A), and Sys1–iRFP preceded GFP–Tlg2 (Fig. 5H–J; Fig. S6C). We also detected a few Sys1–GFP-containing compartments matured into Clc1–mCherry-containing ones (Fig. 5D–G; Fig. S6B), although a vast majority of them were not accompanied by Clc1–mCherry appearance. This is most likely because Clc1–mCherry began to accumulate after the disappearance of GFP–Tlg2 in the same cisterna. Like the Tlg2–Clc1 transition dynamics (Fig. 4E–G), the Sys1–Clc1 transition also progressed via gradual decrease of the area of Sys1-positive zone and complementary increase of the area of Clc1-positive zone (Fig. 5E–G).

We previously reported that Ypt6, the yeast counterpart of Rab6 GTPase regulating tethering and fusion of endosome-derived vesicles, appears at the late Golgi (Suda et al., 2013). We therefore compared the dynamics of Ypt6 versus Sys1 by dual-color 4D SCLIM imaging (Fig. 5K–M; Fig. S7A; Table S1). The temporal dynamics of GFP–Ypt6 and Sys1–iRFP were almost synchronized, consistent with our previous observation that Ypt6 appeared earlier than Sec7 (Suda et al., 2013) (Table S1). Taken together, our present study shows that Sys1 and Ypt6 accumulate prior to Tlg2, Sec7 and Clc1. Our observation that Sys1 appeared before Tlg2 is consistent with the idea that the Sys1–Arl3–Arl1–Imh1 cascade is involved in capturing endosome-derived vesicles to target them to the TGN membrane for Tlg2-mediated fusion.

### COPI accumulates prior to Sys1, Tlg2 and Sec7

**Fig. 6.**
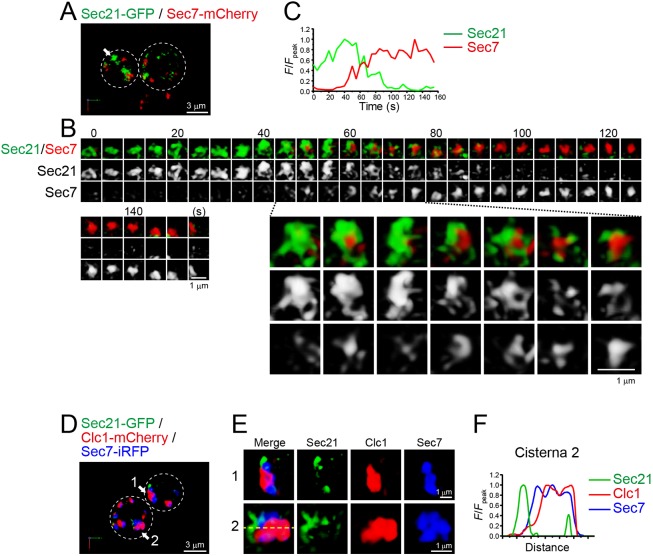
**4D and 3D dynamics of COPI at the Sec7**-**containing** **compartment.** (A–C) Dual-color time-lapse SCLIM imaging of yeast cells expressing Sec21–GFP (a COPI subunit) and Sec7–mCherry. (A) Low-magnification images of the cells. (B) Upper panels show time-lapse images of the single cisterna (the white arrow in A) in the cell. Lower panels show magnified images at the selected time-points. (C) Time course changes in relative fluorescence intensities (*F/F*_peak_) for the green and red channels in B. (D–F) Triple-color SCLIM imaging of yeast cells expressing Sec21–GFP, Clc1–mCherry and Sec7–iRFP. (D) Low-magnification images of the cells. (E) Magnified images of the two cisternae (white arrows in D) in the cells. (F) Line scan analysis of the cisterna 2 fluorescence signal. The *F/F*_peak_ values of green, red and iRFP channels along the yellow broken line in E are profiled. The white broken lines in A,D indicate the edge of the cells. Scale bars; 3 µm (A,D) and 1 µm (B,E).

The TGN and the Golgi can be distinguished by the types of resident coat proteins: the TGN produces clathrin-coated vesicles, whereas the Golgi produces COPI-coated vesicles (Glick and Nakano, 2009; Papanikou and Glick, 2014). The COPI complex consists of seven subunits, Ret1, Sec26, Sec27, Sec21, Ret2, Sec28 and Ret3, and is thought to mediate intra-Golgi and Golgi-to-ER retrograde traffic (Ishii et al., 2016; Papanikou et al., 2015; Jackson, 2014). To examine the transition process from the Golgi to the TGN, we visualized the dynamics of Sec21–GFP versus Sec7–mCherry by dual-color 4D SCLIM (Fig. 6A–C; Fig. S7B; Table S1). Sec7–mCherry began to accumulate at a pre-existing Sec21–GFP-containing compartment, which then gradually increased in volume. As the Sec7–mCherry signal increased, the Sec21–GFP signal decreased and eventually became invisible. During the transition period, the two signals showed complementary mosaic-like distributions (Fig. 6B, lower panels), suggesting the formation of discrete functional zones within a cisterna. We also analyzed Sec21–GFP dynamics versus Clc1–mCherry, but barely observed their colocalization (data not shown). This is probably because Clc1–mCherry began to accumulate after the disappearance of Sec21–GFP in the same cisterna. This finding was further confirmed by triple-color 3D SCLIM imaging of Sec7–iRFP, Clc1–mCherry and Sec21–GFP (Fig. 6D–F; Movie 4). Some Sec7–iRFP-containing compartments harbored both Clc1–mCherry and Sec21–GFP signals, although their spatial distributions were almost completely segregated (Fig. 6F).

**Fig. 7.**
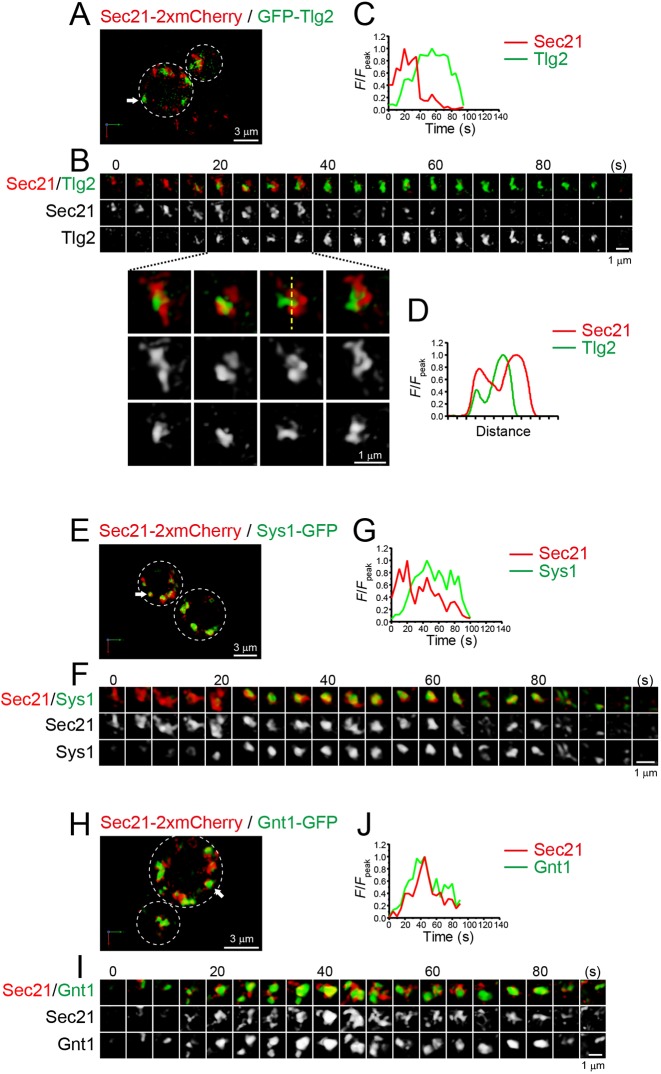
**4D dynamics of COPI subunit Sec21 versus the t-SNARE Tlg2, Sys1 and the glycosylation enzyme Gnt1.** (A–J) Dual-color time-lapse SCLIM imaging of yeast cells expressing Sec21–2×mCherry and GFP–Tlg2 (A–D), Sec21–2×mCherry and Sys1–GFP (E–G), and Sec21–2×mCherry and Gnt1–GFP (H–J). (A,E,H) Low-magnification images of the cells. (B,F,I) Time-lapse images of the single cisternae (white arrows in A, E and H, respectively) in the cells. (C,G,J) Time course changes in relative fluorescence intensities (*F/F*_peak_) for the green and red channels in B, F and I, respectively. (D) Line scan analysis of Sec21–2×mCherry and GFP–Tlg2 signals in the single cisterna. The *F*/*F*_peak_ values (green and red channels) along the yellow broken line are profiled. The white broken lines in A, E and H indicate the edge of the cells. Scale bars: 3 µm (A,E,H) and 1 µm (B,F,I).

We also compared the dynamics of Sec21 tagged with tandem mCherry (2×mCherry) versus GFP–Tlg2 or Sys1–GFP by dual-color 4D SCLIM (Fig. 7A–G; Fig. S7C; Fig. S8A; Table S1). Sec21–2×mCherry appeared prior to GFP–Tlg2 and Sys1–GFP, and line scan analysis showed that the Sec21 and Tlg2 signals were spatially segregated during their transition period (Fig. 7D). These results suggest that the retrograde cargo reception occurs after COPI vesicle formation stage.

In addition to producing COPI vesicles, glycosylation is another indispensable function of the Golgi. Previous reports have shown that Gnt1, an *N*-acetylglucosaminyltransferase, resides at medial-Golgi cisterna (Yoko-o et al., 2003; Ishii et al., 2016). We therefore compared the dynamics of Gnt1–GFP versus Sec21–2×mCherry by dual-color 4D SCLIM (Fig. 7H–J; Fig. S8B; Table S1). The temporal dynamics of Gnt1–GFP and Sec21–2×mCherry were almost synchronized, but their spatial distributions within a cisterna did not completely overlap. This suggests that the two functions of the Golgi, COPI vesicle formation and glycosylation, are executed with the same timing but in different zones.

## DISCUSSION

**Fig. 8.**
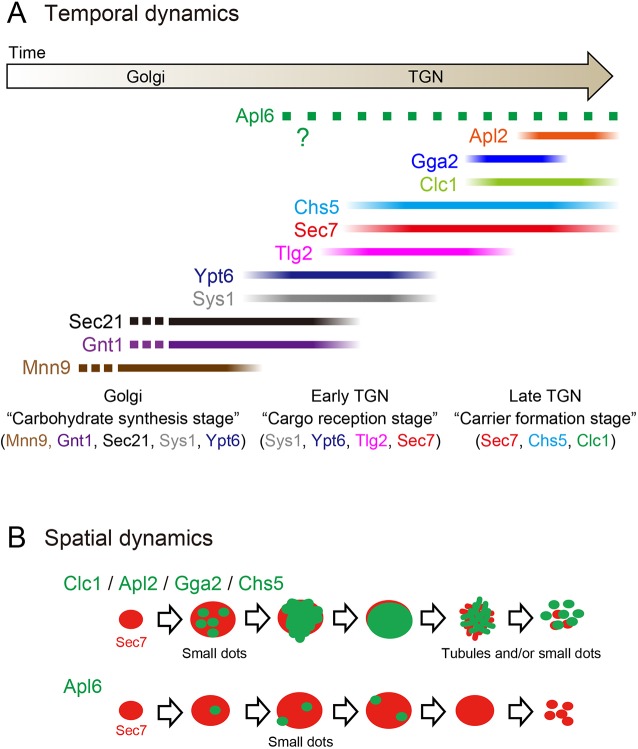
**Schematic presentation of spatiotemporal dynamics of Golgi/TGN-resident proteins.** (A) Temporal dynamics of Golgi/TGN-resident proteins. This schema was drawn based on peak-to-peak duration time (Table S1) obtained in our present and previous (Ishii et al., 2016; Suda et al., 2013) studies. Mnn9, a Golgi mannosyltransferase, is used as a *cis*-Golgi marker (Ishii et al., 2016). The lines show the residence periods of the indicated proteins at a single Golgi/TGN cisterna. All the proteins (solid lines) except Apl6 cover almost entire region of the cisterna, while Apl6 (green dotted line) appears as small dot-like structures on the cisterna. The exact timing of Apl6 appearance and disappearance remained obscure because of high variability in experimental data. The appearance timing of Sec21, Gnt1 and Mnn9 was not examined in the present study (broken lines). Time flows from left to right. We propose that the Golgi-to-TGN transition process is classified into three successive stages: (1) the Golgi stage, when carbohydrate synthesis occurs, which is defined by the presence of Mnn9, Gnt1, Sec21, Sys1 and Ypt6, (2) the early TGN stage when retrograde cargo reception occurs, which is defined by the presence of Sys1, Ypt6, Tlg2 and Sec7, and (3) the late TGN stage, when transport carrier formation occurs, which is defined by the presence of Sec7, Clc1, Apl2, Gga2 and Chs5. (B) Spatial dynamics of coat and adaptor proteins at the TGN. The upper panel shows dynamics of Clc1, Apl2, Gga2 and Chs5 (green) at the Sec7-containing TGN cisterna (red). The coat and adaptors initially appeared as small dot-like structures at the TGN, increased gradually their volume to show signal over entire region of the TGN, and then fragmented into many tubules or small dot-like structures. The lower panel shows the dynamics of Apl6. Apl6 appeared as small dot-like structures but these did not increase their size before they disappeared. See text for details.

In the present study, we performed high-speed and high-resolution 4D live-cell imaging (SCLIM) of a variety of Golgi/TGN-resident proteins in the budding yeast *S. cerevisiae*. Although a part of our findings regarding temporal order of some proteins has already been reported or suggested by other groups (see, for example, Daboussi et al., 2012; Day et al., 2018; McDonold and Fromme, 2014), our advanced microscopy visualized, for the first time, the precise spatial information within a single cisterna. Furthermore, we compared spatiotemporal profiles of 11 different Golgi/TGN-resident proteins under the same experimental conditions. Based on this comprehensive mapping analysis, we propose here that the Golgi–TGN transition process can be classified into the following three successive stages (Fig. 8A). First, the ‘Golgi stage’, defined by the presence of Gnt1 and Sec21, where carbohydrate synthesis and COPI-dependent carrier formation takes place. Second, the ‘early TGN stage’, defined by the presence of Tlg2, where reception of retrograde cargoes occurs. Finally, the ‘late TGN stage’, defined by the presence of Chs5, Clc1, Apl2 and Gga2, where transport carrier formation occurs. Importantly, our SCLIM observations showed that, during the stage transition periods, the earlier and later markers resided simultaneously at a single cisterna in a spatially segregated manner, forming the boundary between the two stages. We also found that, at the late TGN stage, individual coat and adaptor proteins exhibited distinct spatiotemporal assembly dynamics (Fig. 8B), which would contribute to efficient cargo sorting and packaging into different types of carriers.

### Spatial dynamics of coat and adaptor proteins at the TGN

In our SCLIM observations, Clc1, Apl2, Gga2 and Chs5 exhibited similar spatial assembly and disassembly patterns as follows (Fig. 8B). In the beginning, they appeared as multiple small dot-like structures (<0.2 µm in diameter) on the TGN. These dots probably represent clathrin/exomer-coated buds formed on the surface of the TGN. Subsequently, the dots gradually increased their number and size to envelop almost the entire region of the TGN. The accumulated signals were then fenestrated and fragmented into many tubules and/or small dots (<0.2 µm in diameter), which eventually scattered into the cytosol. These structures are reminiscent of what has been previously observed in through electron microscopy of the Golgi/TGN cisterna, with pores and branching buds with a clathrin-like coat (Beznoussenko et al., 2016), and the Clc1-, Apl2- and Gga2-positive small dot-like structures imaged by SCLIM most probably correspond to clathrin-coated buds and/or vesicles.

The dynamics of Apl6 was different from any other adaptors (Fig. 8B). A single TGN cisterna harbored multiple Apl6-positive small dot-like structures (<0.2 µm in diameter), and the Apl6 dots remained stationary for several to a few tens of seconds without changing the size until they disappeared. A previous electron microscopy study showed that the diameter of AP-3-positive vesicles is 50–130 nm (Rehling et al., 1999), consistent with the idea that the observed Apl6-positive dot signals correspond to AP-3-coated buds and vesicles located on and around the TGN. Notably, our 3D colocalization analysis (Fig. S1) showed that only a small portion of the Apl6 signal was present on Sec7-containing TGN cisternae, while the majority was located at Sec7-negative compartments. This suggests that the AP-3 complex mediates not only cargo export at the TGN but also membrane traffic from other organelles, such as endosomes. Indeed, the AP-3 complex resides at endosomal compartments in mammalian cells (Peden et al., 2004; Kent et al., 2012). It has also been reported that, in *S. cerevisiae*, the AP-3 complex is first recruited to the TGN and where it then forms AP-3-coated vesicles that fuse with endocytosed vesicles to become Vps21/Rab5-independent endosomal compartments (Toshima et al., 2014). An alternative hypothesis is that the majority of AP-3 complex might be located at earlier Golgi cisternae and mediate direct cargo transport to the vacuoles independently of the TGN. This hypothesis will be examined carefully in our future work.

### Temporal dynamics of coat and adaptor proteins at the TGN

We found that the coat and adaptor proteins (Clc1, Apl2, Gga2, Chs5 and Apl6) exhibited differential temporal dynamics (Fig. 8A). At the TGN membrane, AP-1, GGA, AP-3 and exomer interact with common molecules such as Arf1-GTP and phosphatidylinositol 4-phosphate (Santiago-Tirado and Bretscher, 2011). On the other hand, they recognize different cargo proteins via specific signal peptide motifs, and different accessory proteins, such as Ent3 for GGA proteins (Daboussi et al., 2012). In addition, recent studies have suggested that the collaborations between different adaptors are important for their assembly and post-Golgi membrane traffic. For example, the timing of AP-1 recruitment to the TGN depends on prior assembly of GGA proteins (Daboussi et al., 2012, 2017). In the fission yeast *Schizosaccharomyces pombe*, the Chs5 homolog Cfr1 interacts with AP-1 and GGA proteins, but not AP-3, and their collaborations are required for post-Golgi traffic (Hoya et al., 2017). Such interplays between different adaptors, cargoes and other TGN-resident molecules (small GTPases, TRAPPs, phospholipids, etc.) (Suda et al., 2018; Guo et al., 2014; Yu and Lee, 2017; Thomas and Fromme, 2016) could contribute to orchestrate their sequential assembly patterns at the TGN, although further biochemical and genetic analyses are required for full understanding of the molecular mechanisms.

### Temporal dynamics of Golgi-TGN transition

Glick's group has recently proposed a three-stage model of Golgi/TGN cisternal maturation (Day et al., 2013; Papanikou and Glick, 2014). In this model, maturing Golgi/TGN cisternae are classified into three successive stages: (1) the ‘cisternal assembly stage’, which includes *cis*-Golgi cisternae; (2) the ‘carbohydrate synthesis stage’, which includes medial- and *trans*-Golgi cisternae; and (3) the ‘carrier formation stage’, which corresponds to the TGN. In the present study, we provided strong experimental evidence to support this model: the carbohydrate synthesis stage, which is defined by the presence of glycosylation enzymes (Gnt1) and COPI (Sec21), and the carrier formation stage, which is defined by the presence of clathrin (Clc1) and exomer (Chs5), exist as spatially and temporally exclusive compartments within a single cisterna (Fig. 8A). Importantly, our study further updated the model such that the TGN stage can be divided into two sub-stages, ‘early TGN’, receiving retrograde traffic and ‘late TGN’, producing transport carriers (Fig. 8A). Based on our updated model, we here propose to redefine previous late Golgi marker proteins. For example, Sec7 should be referred to as a marker for TGN, not for the *trans*-cisterna of the Golgi, and Tlg2 should be classified as a specific marker for early TGN.

At present, the molecular mechanisms underlying the transition from early to late TGN remain largely unknown. One possible mechanism is that Tlg2 recruits Arf1 and Sec7 to the TGN membrane. This hypothesis is based on a previous study that Golgi-targeting motif of Arf1 can bind to membrin, a SNARE protein in mammalian cells (Honda et al., 2005). In plant cells, TGN localization of ARF1 and BIG4, a plant homolog of Sec7, is dependent on ECHIDNA, a plant homolog of the t-SNARE-interacting protein Tvp23 (Jonsson et al., 2017). Another possible mechanism is that Sys1 may contribute to the recruitment of Sec7 and other late TGN components that mediate carrier formation. Sys1 recruits Arl3 and Arl1 (Graham et al., 1994), and Arl1 is known to be involved in the recruitment of Sec7 (McDonold and Fromme, 2014). It is also reported that, in mammalian cells, Arl1 recruits BIG1/2 (also known as ARFGEF1/2), mammalian homologs of Sec7 (Christis and Munro, 2012). Taken together, it is reasonable to suggest that Sys1 and Tlg2 play multiple roles in the TGN functions and maturation: reception of retrograde cargo traffic and recruitment of molecular components required for the late TGN function.

### The TGN in yeast and plant cells

In contrast to dispersed nature of Golgi/TGN cisternae in *S. cerevisiae*, plant cells have tightly stacked Golgi cisternae and two types of TGNs: Golgi-associated TGN (GA-TGN) and Golgi-independent TGN (GI-TGN) (Viotti et al., 2010; Uemura et al., 2014). The GA-TGN is attached onto the *trans*-side of the Golgi stack, while the GI-TGN is segregated from the Golgi stack. Both GI-TGN and GA-TGN harbor plant Tlg2 orthologs, such as SYP41 and SYP43 (Uemura et al., 2014). Electron tomography analyses showed that clathrin-coated buds and secretory buds are more abundant in the GI-TGN than in the GA-TGN (Kang et al., 2011). It is now widely accepted that the plant TGN also behaves in the same manner as early endosomes. The key data supporting this concept is that the lipophilic endocytosis tracer FM4-64 is rapidly incorporated into the TGN before reaching Rab5-positive multivesicular endosomes (Dettmer et al., 2006; Lam et al., 2007; Chow et al., 2008). Recently, it has been reported that the yeast TGN is also the first destination of endocytosed FM4-64 (Day et al., 2018), revealing a striking similarity between the functions of yeast and plant TGNs. Taken together, the main functions and molecular components of the TGN are evolutionarily conserved among species, even though their morphological features are different. Intriguingly, we recently found that the plant TGN regenerates independently of the Golgi stack after transient treatment with brefeldin A (Ito et al., 2017). This suggests that the TGN can be generated not only by Golgi cisternal maturation but also by self-assembly of membrane compartments harboring TGN-resident proteins. This hypothesis should be examined further in future research using other organisms including yeast.

In summary, we demonstrated in the present study the detailed spatiotemporal transition dynamics of the TGN in yeast. We characterized the identity of the TGN as a membrane compartment that is distinct from the Golgi. In the near future, our advanced high-speed and high-resolution imaging techniques, combined with biochemical and genetic analyses, will further provide a vast amount of new information for understanding whole molecular mechanisms underlying Golgi–TGN transition dynamics.

## MATERIALS AND METHODS

### Yeast strains and culture conditions

The yeast *S. cerevisiae* strains and plasmids used in this study are listed in Tables S2–S4. We used the yeast strain YPH499 (Brachmann et al., 1998) and the yeast GFP clone collection (parent strain, BY4741) (Huh et al., 2003). YPH499 *ADE2+* cells were made by integration with pRS402 (Brachmann et al., 1998) digested by *Stu*I into the *ade2* site. Fluorescent proteins used were GFP(S65T) for the green channel, mCherry or tagRFP for the red channel, and iRFP713 (Filonov et al., 2011; Addgene No. 31857) for the far-red channel. Insertion of the fluorescent protein gene into the yeast genome was achieved through PCR-mediated gene replacement (Longtine et al., 1998; Janke et al., 2004) and verified by PCR and fluorescence microscopy. Plasmid-based fluorescent protein-tagged constructs were expressed under the control of the *ADH1* promoter. We confirmed that all the plasmid-integrated cells used in this study can grow normally, and subcellular distribution and temporal dynamics of the labeled proteins were similar to those reported in previous studies.

For microscopy observation, the yeast cells were grown in selective medium (0.67% yeast nitrogen base without amino acids and 2% glucose) with appropriate supplements. The cells were harvested at an early-to-mid logarithmic phase and then seeded on glass coverslips coated with concanavalin A.

### Microscopy

The cells were observed by performing a super-resolution confocal live imaging microscopy (SCLIM) technique that we developed (Kurokawa et al., 2013) at room temperature. The system consists of an inverted microscope (IX73; Olympus) equipped with solid-state lasers with emission at 473 nm (Blues™, 50 mW; Cobolt), 561 nm (Jive™, 50 mW; Cobolt) and 671 nm (CL671-100-S, 100 mW; CrystaLaser), a 100× objective (UPlanSApo, oil, NA 1.4; Olympus), a custom-built piezo actuator (Yokogawa Electric), a high-speed spinning-disk confocal scanner (CSU-10; Yokogawa Electric), a custom-built emission splitter unit, three image intensifiers (Hamamatsu Photonics) with custom-built cooling systems, and three EM-CCD cameras (ImagEM; Hamamatsu Photonics) for green, red and far-red channels. For 3D (*xyz*) observation (Fig. 3G–I; Fig. 6D–F; Fig. S1), 41 optical slices 0.2 μm apart (total *z*-range: 8 µm) were collected at 4 frames/s. For 4D (*xyz* plus time) observation, 21–31 optical slices 0.2 μm apart (total *z*-range: 4–6 µm) were collected at 15 frames/s every 5 s. Z-stack images were converted into 3D voxel data and subjected to deconvolution (iterative restoration) with Volocity software (Perkin Elmer) using a theoretical point-spread function for spinning-disk confocal microscopy. The 3D images were visualized using the ‘3D opacity’ function of Volocity. For time course analyses, cisternae of interest were tracked manually and the fluorescence intensities (*F*) for green, red, or far-red channels were averaged within the region of interest (ROI). To normalize the fluorescence intensity, relative fluorescence over the peak fluorescence (*F*/*F*_peak_) was calculated, where *F*_peak_ was the maximum *F* value during the observation period. For colocalization analysis (Fig. S1), Pearson's correlation coefficient values were calculated using Volocity. The ROI was set to cover a whole single cell and the signal threshold was set by the Costes' method (Costes et al., 2004).

## Supplementary Material

Supplementary information
