# Supplementary material for: Spatiotemporal dissection of the trans-Golgi network in budding yeast
Source: J Cell Sci. 2019 Aug 2;132(15):jcs231159. doi: 10.1242/jcs.231159 (PMC6703704; doi:10.1242/jcs.231159)
Supplement: Supplementary information [file joces-132-231159-s1.pdf]

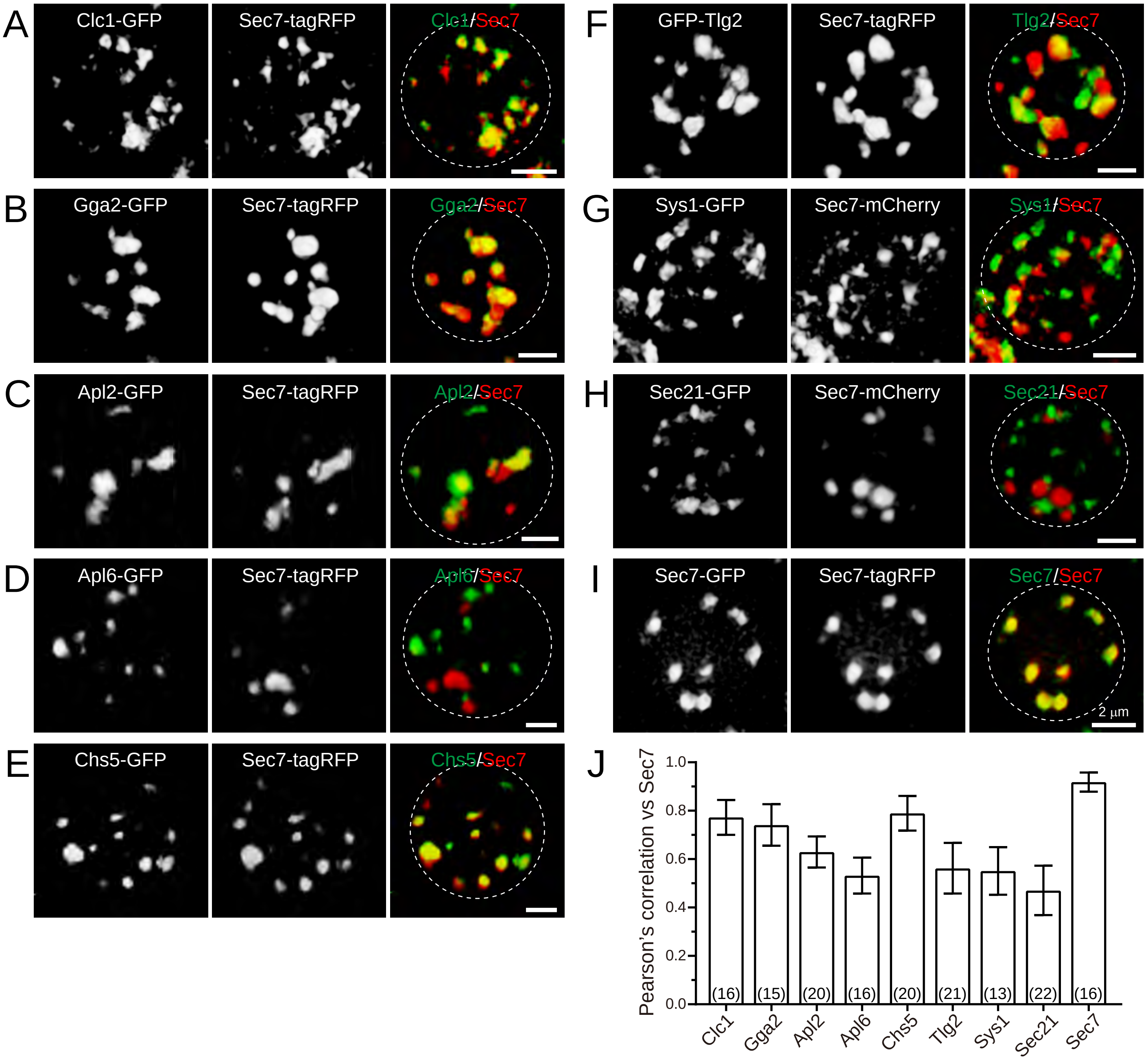

Journal of Cell Science • Supplementary information

Figure S1. 3D co-localization analyses of Golgi/TGN-resident proteins.

(A–H) A variety of GFP-tagged Golgi/TGN-resident proteins (Clc1, Gga2, Apl2, Apl6, Chs5, Tlg2, Sys1, or Sec21) were co-expressed with tagRFP/mCherry-tagged Sec7 and imaged by dual-color 3D SCLIM. **(I)** As a positive control for co-localization analysis, Sec7-GFP and Sec7-tagRFP were co-expressed. Left, center, and right panels show green, red, and overlay channels, respectively. The broken lines indicate the edge of the cells. Scale bars; 2 µm. **(J)** Pearson's correlation coefficient (*r*) for co-localization of the indicated proteins versus Sec7. Numbers in parentheses indicate the number of cells examined. Each value represents mean ± standard deviation (SD).

Journal of Cell Science • Supplementary information

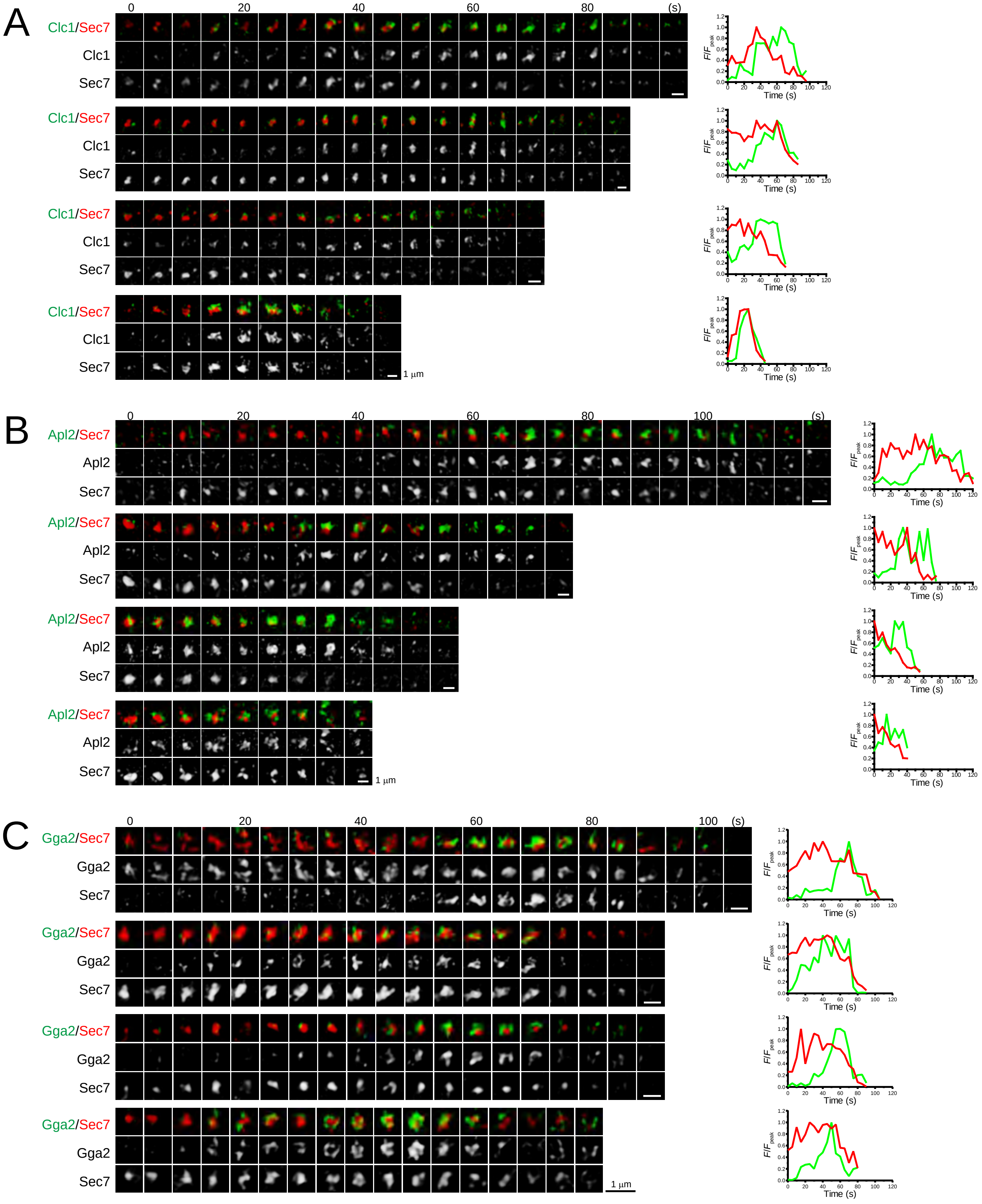

Journal of Cell Science • Supplementary information

Figure S2. 4D dynamics of Clc1, Apl2, and Gga2 versus Sec7.

(A) Clc1-GFP versus Sec7-tagRFP. **(B)** Apl2-GFP versus Sec7-tagRFP. **(C)** Gga2-GFP versus Sec7-tagRFP. In relation to **Fig. 1A–I**, time-lapse images of four cisternae and time course changes in their fluorescence intensities (F/F_{peak}) are shown. Scale bars; 1 µm.

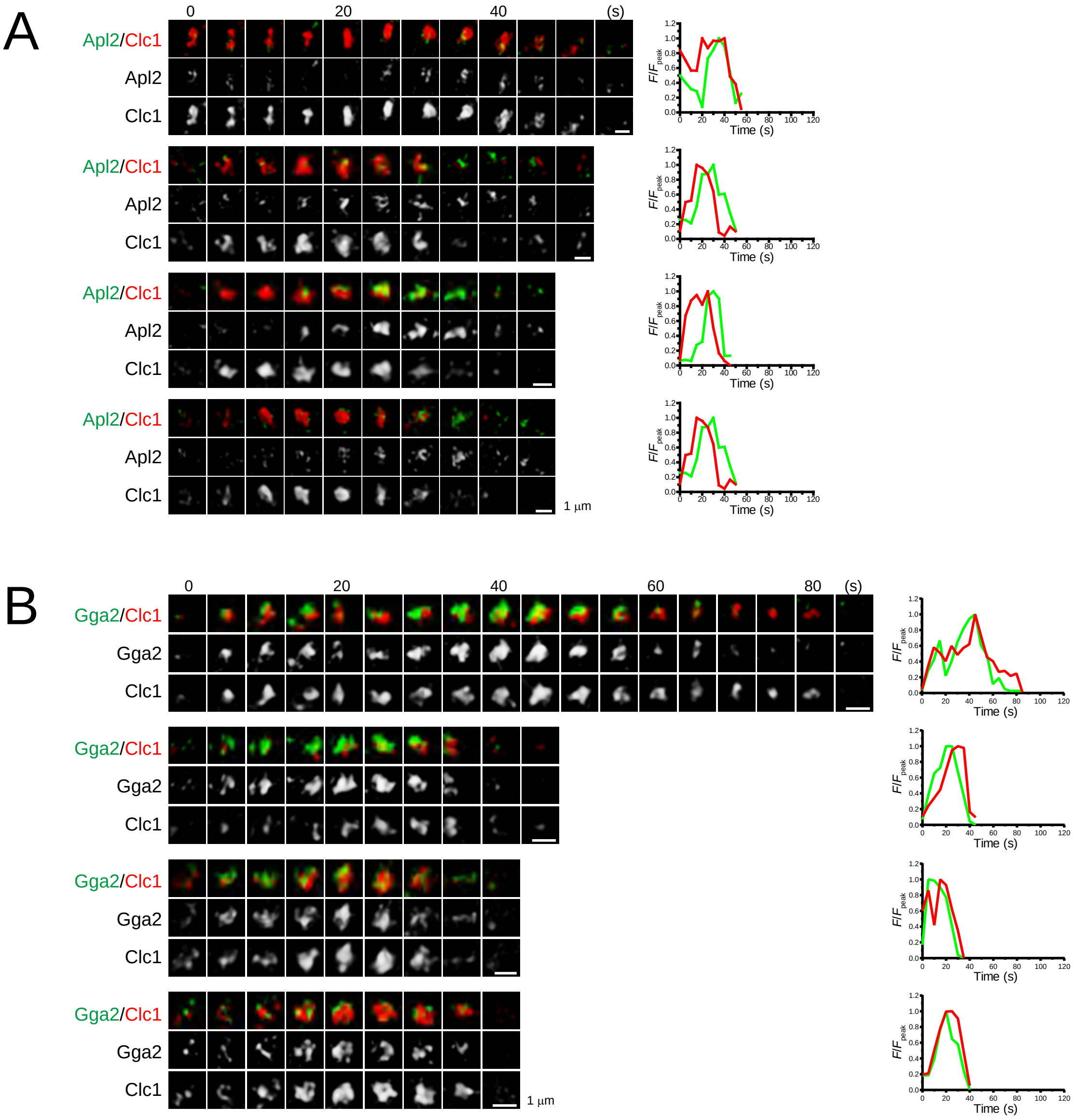

Figure S3. 4D dynamics of Apl2 and Gga2 versus Clc1.

(A) Apl2-GFP versus Clc1-mCherry. **(B)** Gga2-GFP versus Clc1-mCherry. In relation to **Fig. 1J–O**, time-lapse images of four cisternae and time course changes in their fluorescence intensities (F/F_{peak}) are shown. Scale bars; 1 μm.

Journal of Cell Science • Supplementary information

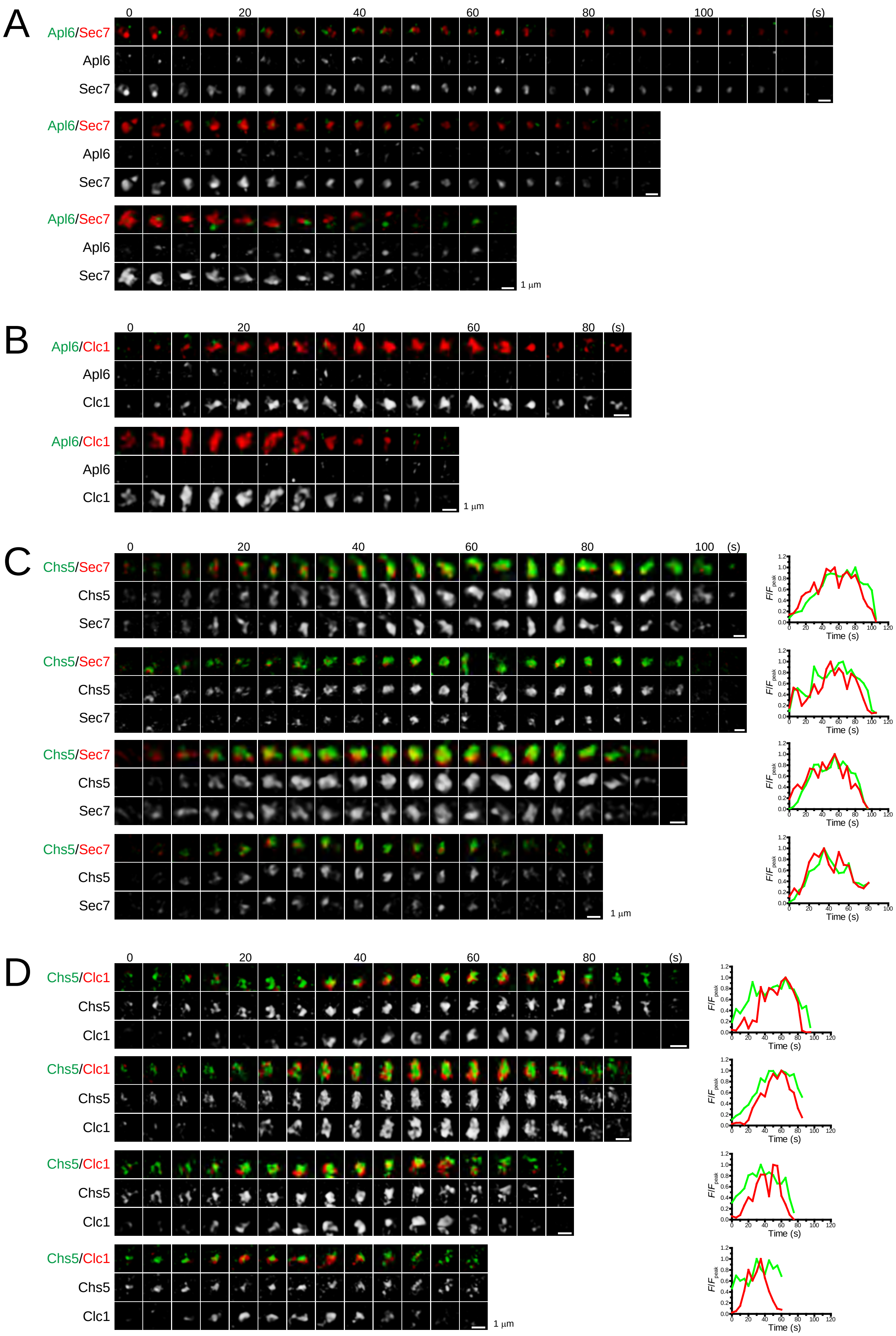

Journal of Cell Science • Supplementary information

Figure S4. 4D dynamics of Apl6 and Chs5 versus Sec7 and Clc1.

(A) Apl6-GFP versus Sec7-tagRFP. **(B)** Apl6-GFP versus Clc1-mCherry. **(C)** Chs5-GFP versus Sec7-tagRFP. **(D)** Chs5-GFP versus Clc1-mCherry. In relation to **Figs. 2 and 3A–F**, time-lapse images of 2-4 cisternae and time course changes in their fluorescence intensities (F/F_{peak}) are shown. Scale bars; 1 µm.

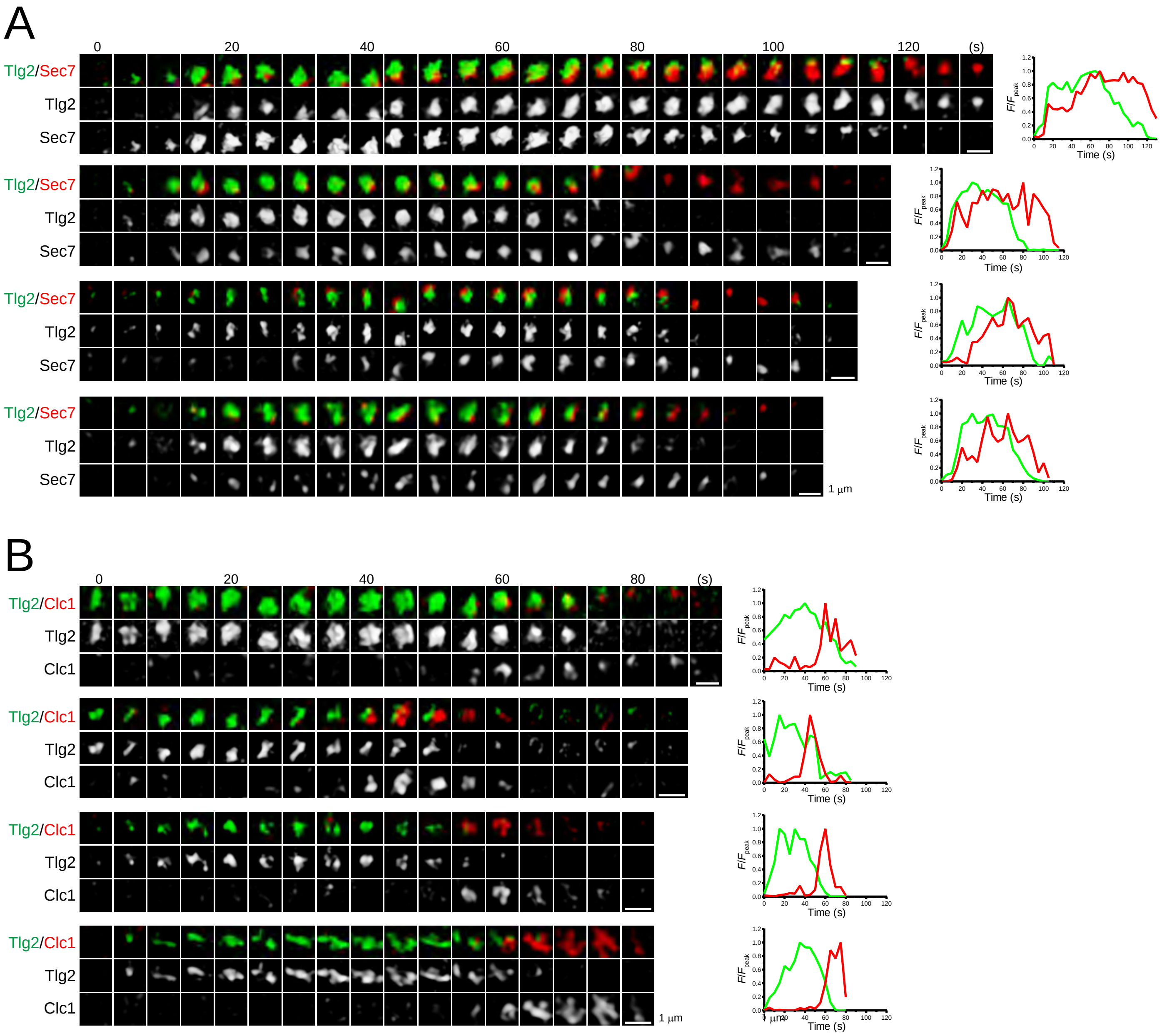

Figure S5. 4D dynamics of Tlg2 versus Sec7 and Clc1.

(A) GFP-Tlg2 versus Sec7-tagRFP. **(B)** Tlg2-GFP versus Clc1-mCherry. In relation to **Fig. 4**, time-lapse images of four cisternae and time course changes in their fluorescence intensities (F/F_{peak}) are shown. Scale bars; 1 μm.

Journal of Cell Science • Supplementary information

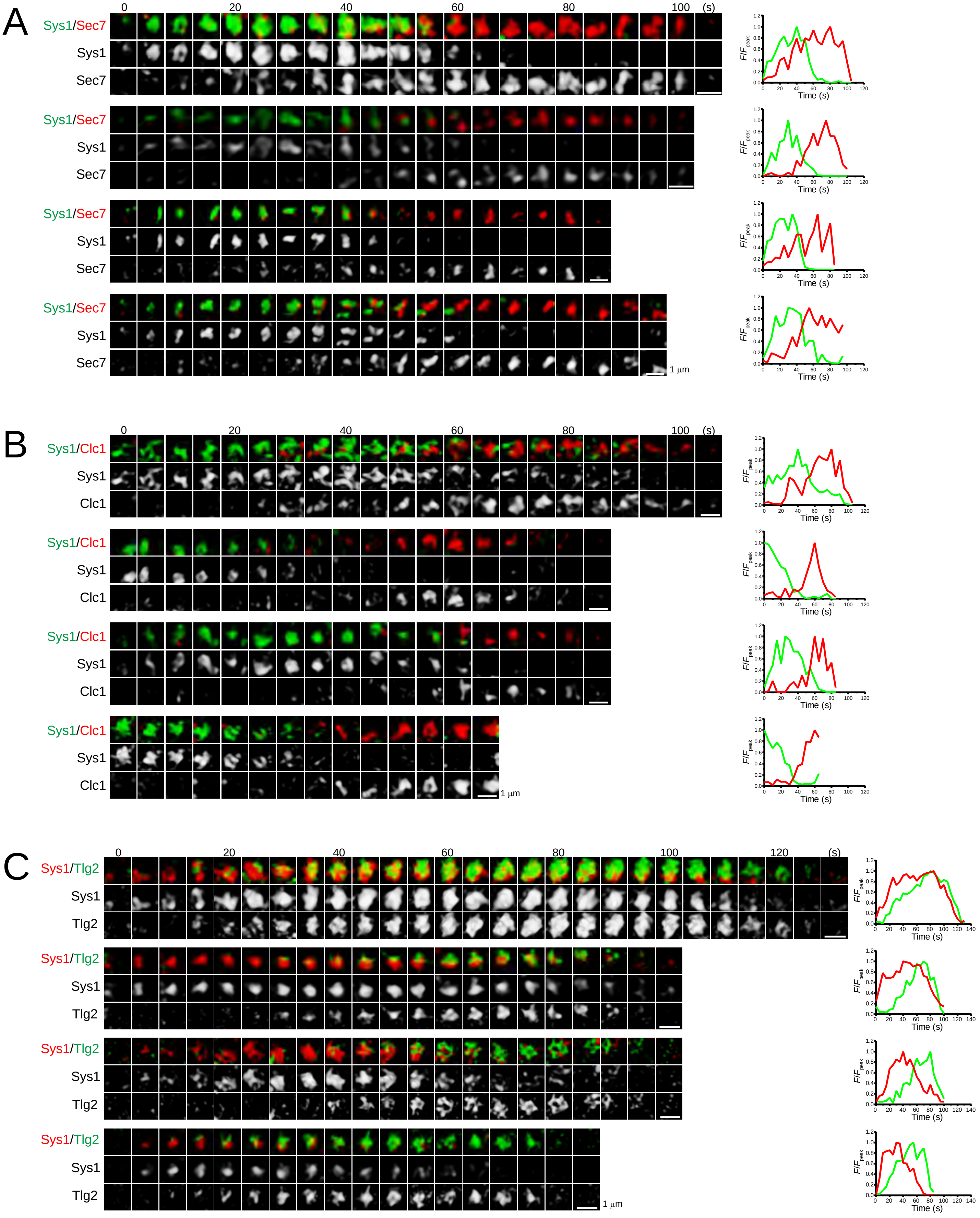

Journal of Cell Science • Supplementary information

Figure S6. 4D dynamics of Sys1 versus Sec7, Clc1, and Tlg2.

(A) Sys1-GFP versus Sec7-mCherry. **(B)** Sys1-GFP versus Clc1-mCherry. **(C)** Sys1-iRFP versus GFP-Tlg2. In relation to **Fig. 5A–J**, time-lapse images of four cisternae and time course changes in their fluorescence intensities (F/F_{peak}) are shown. Scale bars; 1 µm.

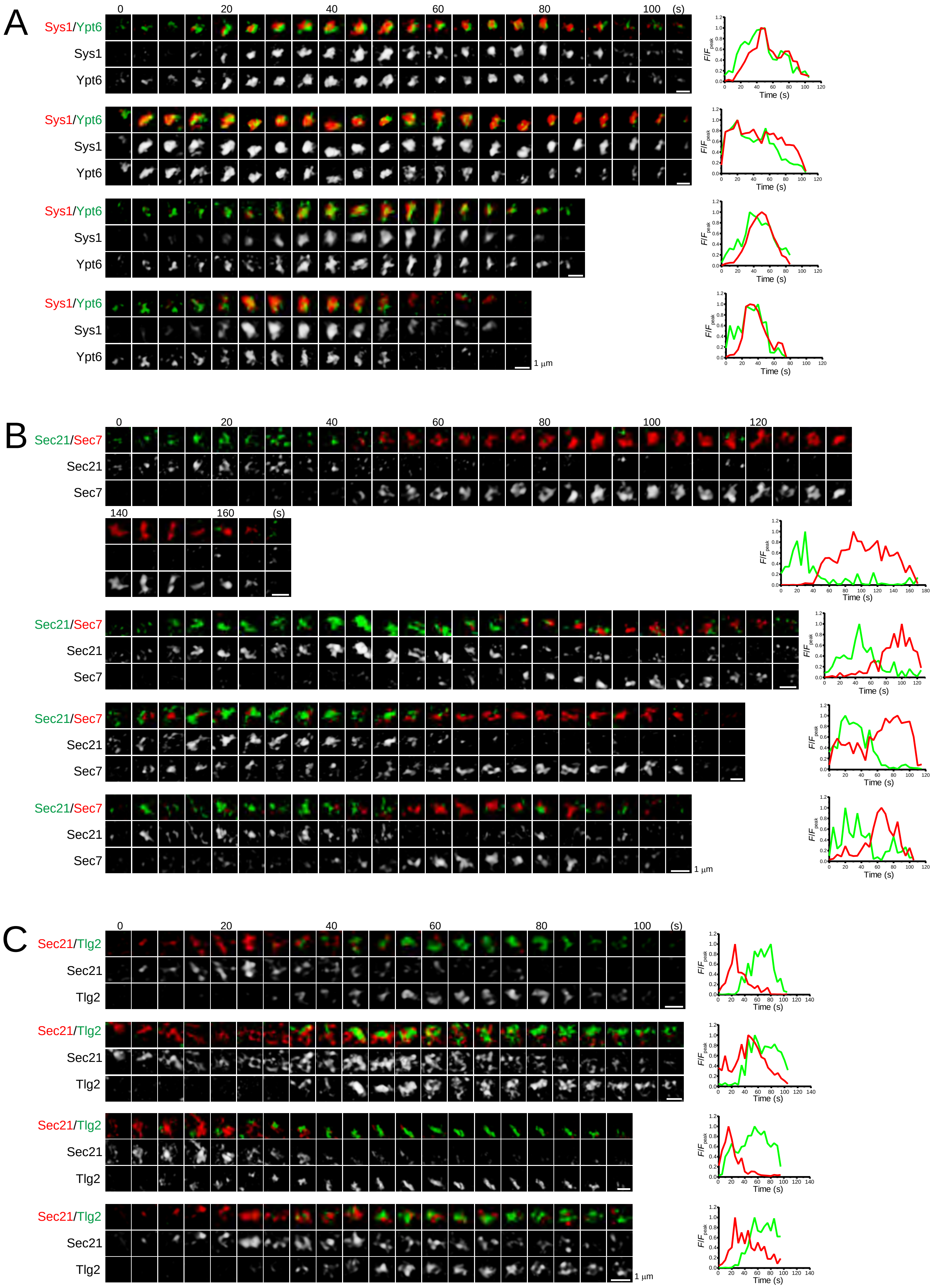

Journal of Cell Science • Supplementary information

Figure S7. 4D dynamics of Sys1 versus Ypt6, Sec21 versus Sec7, and Sec21 versus Tlg2.

(A) Sys1-iRFP versus GFP-Ypt6. **(B)** Sec21-GFP versus Sec7-mCherry. **(C)** Sec21-2xmCherry versus GFP-Tlg2. In relation to **Fig. 5K–M**, **Fig. 6A–C**, **and Fig. 7A–D**, time-lapse images and four cisternae and time course changes in their fluorescence intensities (F/F_{peak}) are shown. Scale bars; 1 µm.

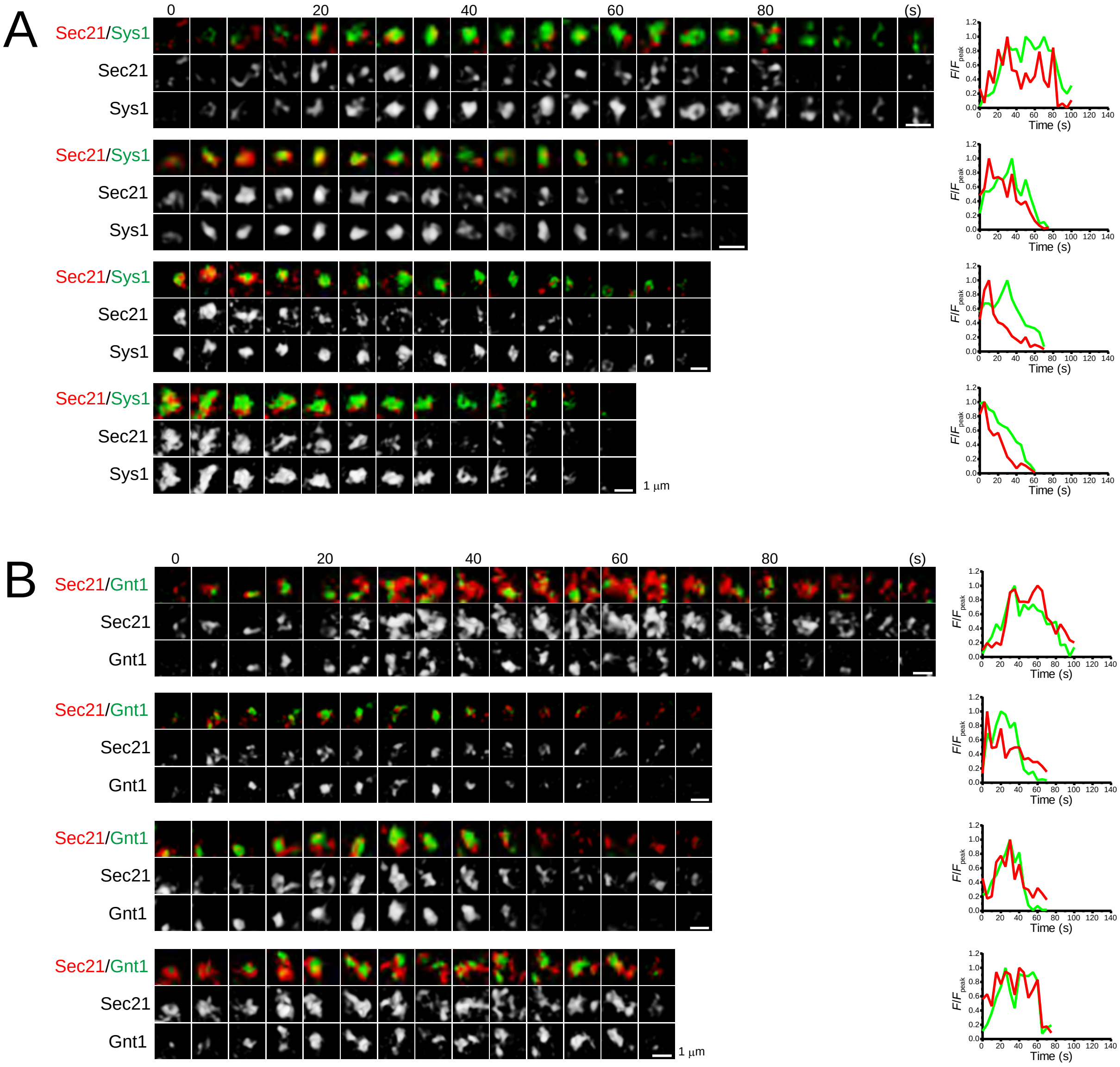

Figure S8. 4D dynamics of Sec21 versus Sys1 and Gnt1.

(A) Sec21-2xmCherry versus Sys1-GFP. **(B)** Sec21-2xmCherry versus Gnt1-GFP. In relation to **Fig. 7E–J**, time-lapse images of four cisternae and time course changes in their fluorescence intensities (F/F_{peak}) are shown. Scale bars; 1 μm.

Table S1. Peak-to-peak duration time.

No.	Labeled proteins	Peak-to-peak duration (s) (mean ± SD)	‡Number of puncta (n) in total cells (N) n	N
1	Sec7-tagRFP → Clc1-GFP	21.0 ± 11.9	5	5
2	Sec7-tagRFP → Apl2-GFP	25.0 ± 15.8	6	5
3	Sec7-tagRFP → Gga2-GFP	21.7 ± 15.8	9	8
4	Clc1-mCherry → Apl2-GFP	14.0 ± 6.2	10	7
5	Gga2-GFP → Clc1-mCherry	2.3 ± 5.0	14	8
6	Sec7-tagRFP → Chs5-GFP	8.0 ± 11.5	5	3
7	Chs5-GFP → Clc1-mCherry	3.3 ± 6.1	6	4
8	GFP-Tlg2 → Sec7-tagRFP	27.5 ± 20.6	14	7
9	GFP-Tlg2 → Clc1-mCherry	30.4 ± 17.2	11	9
10	Sys1-GFP → Sec7-mCherry	31.4 ± 16.0	11	8
11	Sys1-iRFP → GFP-Tlg2	25.8 ± 23.2	13	9
*12	GFP-Ypt6 → Sec7-mRFP	47.9 ± 17.7	9	6
13	GFP-Ypt6 → Sys1-iRFP	2.2 ± 13.4	16	10
14	Sec21-GFP → Sec7-mCherry	67.9 ± 24.5	12	8
15	Sec21-2xmCherry → GFP-Tlg2	33.2 ± 19.8	11	6
16	Sec21-2xmCherry → Sys1-GFP	9.5 ± 13.0	10	7
17	Gnt1-GFP → Sec21-2xmCherry	0.6 ± 14.0	9	7
†18	Mnn9-mCherry → Gnt1-GFP	38.6 ± 11.6	17	14
†19	Mnn9-mCherry → Sys1-GFP	45.6 ± 19.6	13	8
†20	mRFP-Sed5 → Sec7-GFP	87.8 ± 34.0	11	10

*Suda, Y., Kurokawa, K., Hirata, R., and Nakano, A. 2013. Rab GAP cascade regulates dynamics of Ypt6 in the Golgi traffic. *Proc. Natl. Acad. Sci. U S A.* 110:18976-18981.

†Ishii, M., Suda, Y., Kurokawa, K., and Nakano, A. 2016. COPI is essential for Golgi cisternal maturation and dynamics. *J. Cell Sci.*129:3251-3261.

‡Number of puncta and cells used for the calculation of peak-to-peak duration. In each cell, 1 - 3 puncta were selected for the calculation.

Journal of Cell Science • Supplementary information

Table S2. Yeast strains used in this study.

Strain	Genotype	Source
YPH499	*MAT**a** ura3-52 lys2-801 ade2-101 trp1-Δ63 his3-Δ200 leu2-Δ1*	1
BY4741	*MAT**a** his3Δ1 leu2Δ0 met15Δ0 ura3Δ0*	2
43-C1	BY4741 *CLC1-GFP(S65T)::HIS3MX6*	3
11-D11	BY4741 *GGA2-GFP(S65T)::HIS3MX6*	3
38-E4	BY4741 *APL6-GFP(S65T)::HIS3MX6*	3
YSY1	YPH499 *ADE2::pRS402 CLC1-GFP(S65T)::HIS3MX6*	This study
YSY112	YPH499 *ADE2::pRS402 SEC7-GFP(S65T)::kanMX6*	This study
YSY115	YPH499 *ADE2::pRS402 SEC7-mCherry::natNT2*	This study
TTY3	BY4741 *APL6-GFP(S65T)::HIS3MX6 CLC1-mCherry::natNT2*	This study
TTY4	YPH499 *ADE2::pRS402 CLC1-mCherry::natNT2*	This study
TTY5	YPH499 *ADE2::pRS402 APL2-GFP(S65T)::HIS3MX6*	This study
TTY6	YPH499 *ADE2::pRS402 APL6-GFP(S65T)::HIS3MX6*	This study
TTY10	YPH499 *ADE2::pRS402 CHS5-GFP(S65T)::HIS3MX6*	This study
TTY11	YPH499 *ADE2::pRS402 CLC1-mCherry::natNT2 APL2-GFP(S65T)::HIS3MX6*	This study
TTY20	YPH499 *ADE2::pRS402 CLC1-mCherry::natNT2 APL6-GFP(S65T)::HIS3MX6*	This study
TTY21	YPH499 *ADE2::pRS402 CLC1-mCherry::natNT2 CHS5-GFP(S65T)::HIS3MX6*	This study
TTY24	YPH499 *ADE2::pRS402 CLC1-mCherry::natNT2 SEC21-GFP(S65T)::HIS3MX6*	This study
TTY27	YPH499 *ADE2::pRS402 CLC1-mCherry::natNT2 GGA2-GFP(S65T)::HIS3MX6*	This study
TTY29	YPH499 *ADE2::pRS402 SEC21-2xmCherry::hphNT1*	This study

1. Sikorski, R.S., and Hieter, P. 1989. A system of shuttle vectors and yeast host strains designed for efficient manipulation of DNA in *Saccharomyces cerevisiae. Genetics*. 122:19-27.
2. Brachmann, C.B., Davies, A., Cost, G.J., Caputo, E., Li, J., Hieter, P., and Boeke, J.D. 1998. Designer deletion strains derived from *Saccharomyces cerevisiae* S288C: a useful set of strains and plasmids for PCR-mediated gene disruption and other applications. *Yeast*. 14:115-132.
3. Huh, W.K., Falvo, J.V., Gerke, L.C., Carroll, A.S., Howson, R.W., Weissman, J.S., and O'Shea, E.K. 2003. Global analysis of protein localization in budding yeast. *Nature*. 425:686-691.

Journal of Cell Science • Supplementary information

Table S3. Plasmids used in this study.

Plasmid name	Description	Source
pRS402	*ADE2*, integration	1
pFA6a-GFP(S65T)-HIS3MX6	PCR template	2
pFA6a-mCherry-natNT2	PCR template	3
pFA6a-2xmCherry-hphNT1	PCR template	4
pRS316-ADH1p-SEC7-tagRFP	*URA3*, CEN, *ADH1p-SEC7-tagRFP*	This study
pRS316-ADH1p-SEC7-iRFP713	*URA3*, CEN, *ADH1p-SEC7-iRFP*	This study
pRS314-ADH1p-GFP(S65T)-TLG2	*TRP1*, CEN, *ADH1p-GFP-TLG2*	This study
pRS314-ADH1p-SYS1-GFP(S65T)	*TRP1*, CEN, *ADH1p-SYS1-GFP*	5
pRS316-ADH1p-SYS1-iRFP713	*URA3*, CEN, *ADH1p-SYS1-iRFP*	6
pRS314-ADH1p-YPT6-GFP(S65T)	*TRP1*, CEN, *ADH1p-YPT6-GFP*	7
pRS316-ADH1p-SEC21-GFP(S65T)	*URA3*, CEN, *ADH1p-SEC21-GFP*	This study
pRS316-ADH1p-GNT1-GFP(S65T)	*URA3*, CEN, *ADH1p-GNT1-GFP*	5

1. Brachmann, C.B., Davies, A., Cost, G.J., Caputo, E., Li, J., Hieter, P., and Boeke, J.D. 1998. Designer deletion strains derived from *Saccharomyces cerevisiae* S288C: a useful set of strains and plasmids for PCR-mediated gene disruption and other applications. *Yeast*. 14:115-132.
2. Longtine, M.S., McKenzie, A. 3rd, Demarini, D.J., Shah, N.G., Wach, A., Brachat, A., Philippsen, P., and Pringle, J.R. 1998. Additional modules for versatile and economical PCR-based gene deletion and modification in *Saccharomyces cerevisiae*. *Yeast*. 14:953-961.
3. Kurokawa, K., Okamoto, M., and Nakano, A. 2014. Contact of *cis*-Golgi with ER exit sites executes cargo capture and delivery from the ER. *Nat. Commun*. 5:3653.
4. Suzuki, K., Akioka, M., Kondo-Kakuta, C., Yamamoto, H., and Ohsumi, Y. 2013. Fine mapping of autophagy-related proteins during autophagosome formation in *Saccharomyces cerevisiae*. *J. Cell Sci*. 126:253425-253444.
5. Ishii, M., Suda, Y., Kurokawa, K., and Nakano, A. 2016. COPI is essential for Golgi cisternal maturation and dynamics. *J. Cell Sci*.129:3251-3261.
6. Kurokawa, K., Osakada, H., Kojidani, T., Waga, M., Suda, Y., Asakawa, H., Haraguchi, T., and Nakano, A. 2019. Visualization of secretory cargo transport within the Golgi apparatus. *J. Cell Biol*. 218:1602-1618.
7. Suda, Y., Kurokawa, K., Hirata, R., and Nakano, A. 2013. Rab GAP cascade regulates dynamics of Ypt6 in the Golgi traffic. *Proc. Natl. Acad. Sci. U S A*. 110:18976-18981.

Journal of Cell Science • Supplementary information

Table S4. Strains and plasmids used in each experiment.

Experiment (Figure number)	Strain(s) and plasmid(s)
Fig. 1A-C, S1A, S2A	YSY1 with pRS316-ADH1p-SEC7-tagRFP, 43-C1 with pRS316-ADH1p-SEC7-tagRFP
Fig. 1D-F, S1C, S2B	TTY5 with pRS316-ADH1p-SEC7-tagRFP
Fig. 1G-I, S1B, S2C	11-D11 with pRS316-ADH1p-SEC7-tagRFP
Fig. 1J-L, S3A	TTY11
Fig. 1M-O, S3B	TTY27
Fig. 2A,B, S1D, S4A	TTY6 with pRS316-ADH1p-SEC7-tagRFP
Fig. 2C,D, S4B	TTY3, TTY20
Fig. 3A-C, S1E, S4C	TTY10 with pRS316-ADH1p-SEC7-tagRFP
Fig. 3D-F, S4D	TTY21
Fig. 3G-I	TTY21 with pRS316-ADH1p-SEC7-iRFP
Fig. 4A-C, S1F, S5A	YPH499 *ADE2::pRS402* with pRS314-ADH1p-GFP-TLG2 and pRS316-ADH1p-SEC7-tagRFP
Fig. 4D-G, S5B	TTY4 with pRS314-ADH1p-GFP-TLG2
Fig. 5A-C, S1G, S6A	YSY115 with pRS314-ADH1p-SYS1-GFP
Fig. 5D-G, S6B	TTY4 with pRS314-ADH1p-SYS1-GFP
Fig. 5H-J, S6C	YPH499 *ADE2::pRS402* with pRS314-ADH1p-GFP-TLG2 and pRS316-ADH1p-SYS1-iRFP
Fig. 5K-M, S7A	YPH499 *ADE2::pRS402* with pRS314-ADH1p-YPT6-GFP and pRS316-ADH1p-SYS1-iRFP
Fig. 6A-C, S1H, S7B	YSY15 with pRS316-ADH1p-SEC21-GFP
Fig. 6D-F	TTY24 with pRS316-ADH1p-SEC7-iRFP
Fig. 7A-D, S7C	TTY29 with pRS314-ADH1p-GFP-TLG2
Fig. 7E-G, S8A	TTY29 with pRS314-ADH1p-SYS1-GFP
Fig. 7H-J, S8B	TTY29 with pRS316-ADH1p-GNT1-GFP
Fig. S1I	YSY112 with pRS316-ADH1p-SEC7-tagRFP

Journal of Cell Science • Supplementary information

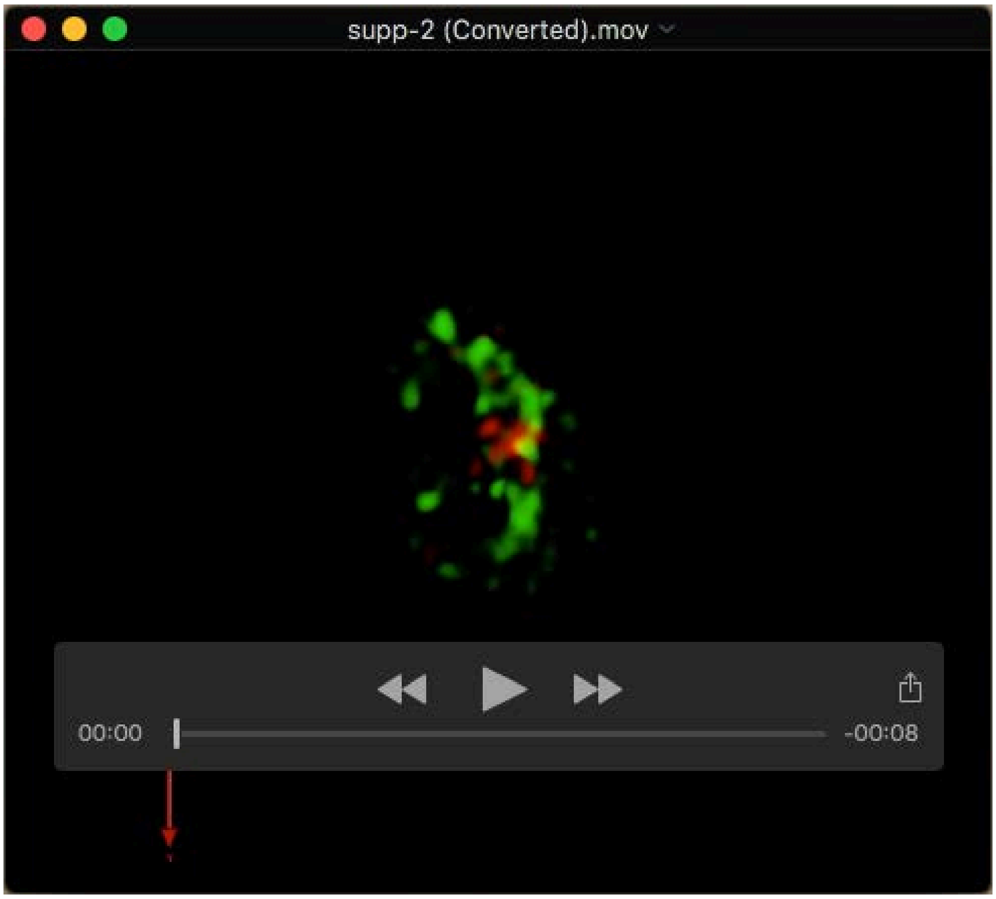

Movie 1. Spatial distributions of Clc1-GFP (green) and Sec7-tagRFP (red) at a cisterna (**Fig. 1B,** time-point of 65 s).

Journal of Cell Science • Supplementary information

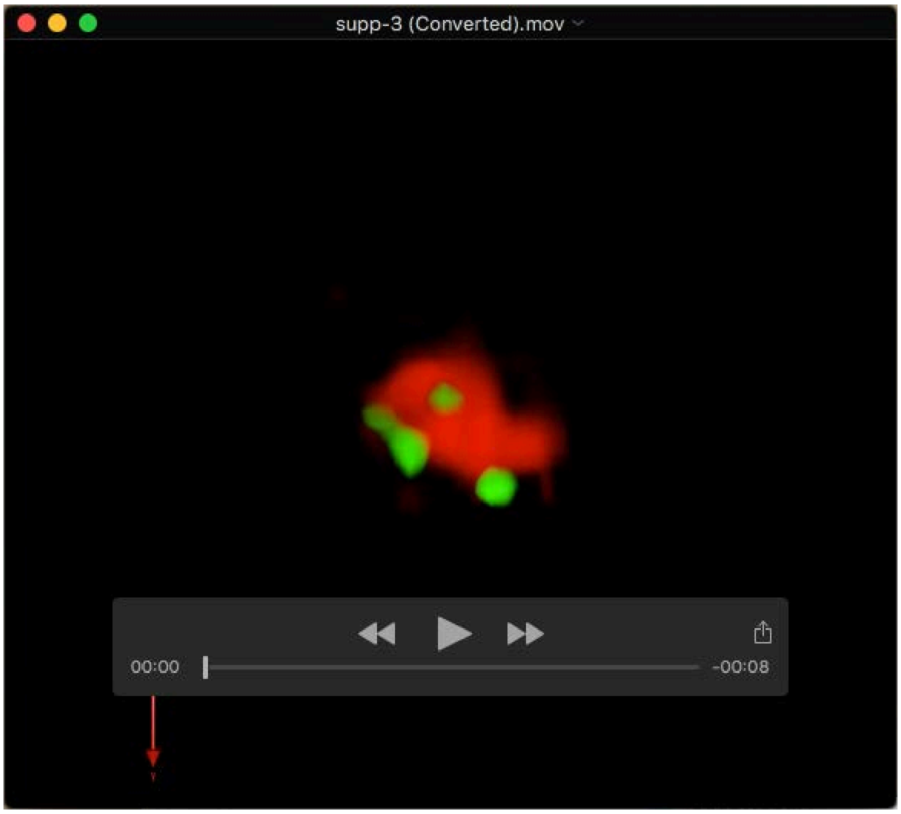

Movie 2. Spatial distributions of Apl6-GFP (green) and Sec7-tagRFP (red) at a cisterna (**Fig. 2B,** time-point of 35 s).

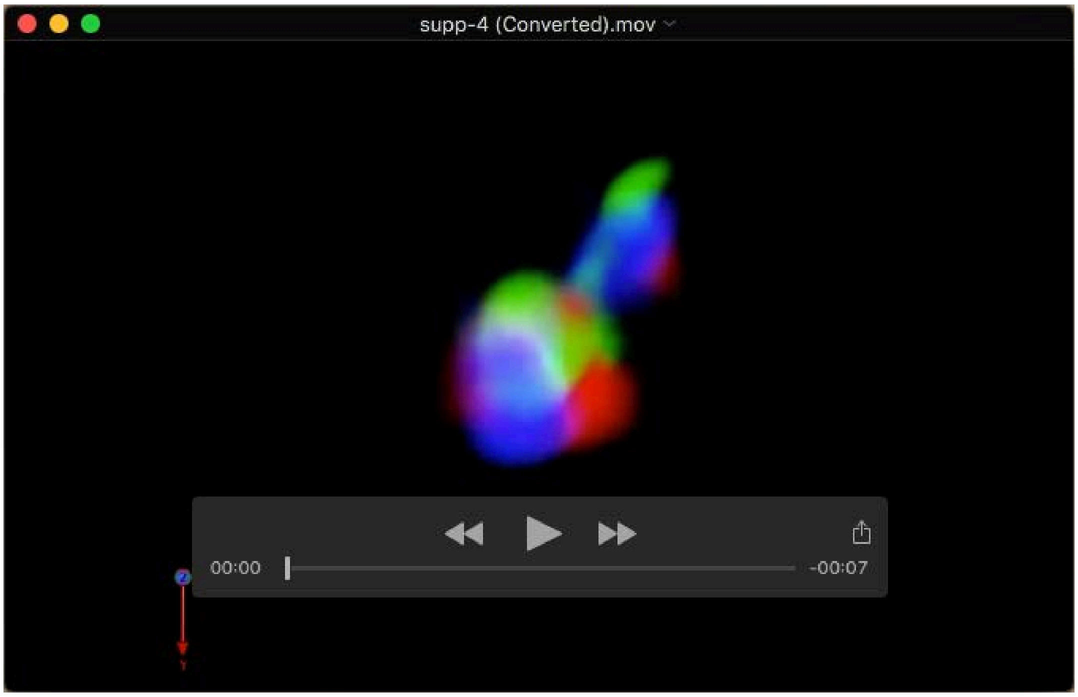

Movie 3. Spatial distributions of Chs5-GFP (green), Clc1-mCherry (red), and Sec7-iRFP (blue) at a cisterna (**Fig. 3H,** cisterna 2).

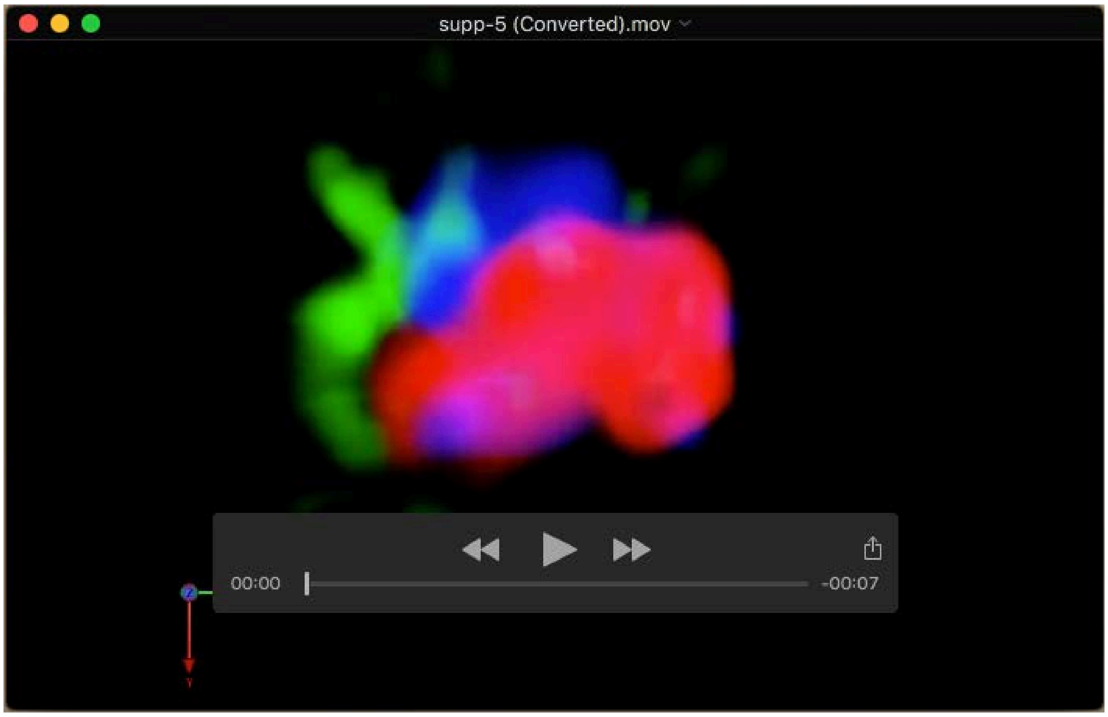

Movie 4. Spatial distributions of Sec21-GFP (green), Sec7-iRFP (blue), and Clc1-mCherry (red) at a cisterna (**Fig. 6E,** cisterna 2).

Journal of Cell Science • Supplementary information
